# Cell fate determined by the activation balance between PKR and SPHK1

**DOI:** 10.1038/s41418-020-00608-8

**Published:** 2020-08-15

**Authors:** Han Qiao, Tianqing Jiang, Peiqiang Mu, Xiaoxuan Chen, Xianhui Wen, Zhangsheng Hu, Shulin Tang, Jikai Wen, Yiqun Deng

**Affiliations:** 1 Guangdong Provincial Key Laboratory of Protein Function and Regulation in Agricultural Organisms, College of Life Sciences, South China Agricultural University, Guangzhou, 510642 Guangdong PR China; 2 Key Laboratory of Zoonosis of Ministry of Agriculture and Rural Affairs, South China Agricultural University, Guangzhou, 510642 Guangdong PR China; 3 Guangdong Laboratory for Lingnan Modern Agriculture, South China Agricultural University, Guangzhou, 510642 Guangdong PR China

**Keywords:** Kinases, G protein-coupled receptors

## Abstract

Double-stranded RNA (dsRNA)-dependent protein kinase R (PKR) activation via autophosphorylation is the central cellular response to stress that promotes cell death or apoptosis. However, the key factors and mechanisms behind the simultaneous activation of pro-survival signaling pathways remain unknown. We have discovered a novel regulatory mechanism for the maintenance of cellular homeostasis that relies on the phosphorylation interplay between sphingosine kinase 1 (SPHK1) and PKR during exogenous stress. We identified SPHK1 as a previously unrecognized PKR substrate. Phosphorylated SPHK1, a central kinase, mediates the activation of PKR-induced pro-survival pathways by the S1P/S1PR1/MAPKs/IKKα signal axis, and antagonizes PKR-mediated endoplasmic reticulum (ER) stress signal transduction under stress conditions. Otherwise, phosphorylated SPHK1 also acts as the negative feedback factor, preferentially binding to the latent form of PKR at the C-terminal kinase motif, inhibiting the homodimerization of PKR, suppressing PKR autophosphorylation, and reducing the signaling strength for cell death and apoptosis. Our results suggest that the balance of the activation levels between PKR and SPHK1, a probable hallmark of homeostasis maintenance, determines cell fate during cellular stress response.

## Introduction

Normally there is an equilibrium between the net growth rate and the net death rate of cells, but this physiological homeostasis alters upon exposure to various extracellular and intracellular stress factors [1]. Adaptive cellular responses arise in response to stressful stimuli. Such responses involve manifold changes in a complex array of proteins with apoptotic or pro-survival properties. In most cases, overloaded stress signals lead to the activation of cell death-associated cascades [2]. Among these pro-apoptotic stress sensors, double-stranded RNA (dsRNA)-activated protein kinase (PKR) has been identified as an essential upstream signal that allows cells to respond adequately to various stress signals including dsRNA, pro-inflammatory factors, growth factors, cytokines, mycotoxins, and oxidative stress, and they mediate cell growth control, apoptosis, and other physiological changes in cellular behavior [3–6].

As a member of the alpha subunit of eukaryotic initiation factor 2 (eIF2α)-specific kinase subfamily, PKR was initially characterized as a core translational inhibitor in an antiviral process regulated by interferons [7]. In addition to the C-terminal kinase catalytic domain shared by other eIF2α kinases, PKR has an N-terminal dsRNA-binding domain (dsRBD) that regulates its activity. As a serine/threonine kinase, two distinct kinase activities were characterized: autophosphorylation through homodimerization [8], and phosphorylation of eIF2α at Ser51, which inhibits protein synthesis [9]. In non-stressed cells, PKR exists in a monomeric latent state owing to its dsRBD, which acts as an autoinhibitory motif and occludes the activation segment of the kinase. During stress conditions, PKR triggers homodimerization by relieving its autoinhibitory form and undergoing rapid autophosphorylation in a stretch of amino acids termed as the activation site. The phosphorylation of residues Thr446 and Thr451 in the C-terminal activation loop of PKR is considered as the biomarker for its autophosphorylation and activation, both in vitro and in vivo [10]. Substitution of Thr451 with alanine annuls PKR function and prevents substrate recognition. Moreover, Thr451 phosphorylation stabilizes PKR dimerization, which in turn increases the catalytic activity of the kinase [11].

PKR kinase activity is regulated in various ways in cells when experiencing stress stimuli or viral infection. The PKR activator protein (PACT) and its orthologue PKR-associated protein X (RAX) act as physiological activators through direct protein–protein interactions during various cell stress stimuli [12, 13]. Several cellular or viral proteins have been identified that are able to inhibit PKR kinase activity, either by direct interaction, or by competitively binding to PKR activators or substrates [14**–**18]. The myriad ways by which viruses inhibit PKR function have been investigated to determine how they escape. However, there have been few studies on the regulation of PKR enzyme activity by responsive intracellular proteins during stress response.

Besides the pro-apoptotic pathway, cells under stress conditions must elicit appropriate protective responses. Essentially, if a stress stimulus does not exceed a certain threshold, the cells survive by mounting pro-survival strategies to defend themselves and recover from the insult [2]. The pro-survival property of sphingosine kinase 1 (SPHK1) has been extensively investigated in response to several stimuli such as the growth factors PDGF, EGF, NGF, and two stress indicators, lipopolysaccharides (LPS) and tumor necrosis factor-α (TNF‐α) [19**–**23]. Phosphorylation at Ser225 is necessary for the activation of SPHK1 and its translocation to the plasma membrane [24]. This leads to the production of sphingosine-1-phosphate (S1P), a signaling messenger implicated in various diseases including cancer, atherosclerosis, and metabolic disorders [25]. The generated S1P is then exported from cells via S1P transporters, such as SPNS2 and ABCC1 [26]. Extracellular S1P stimulates any of five specific G protein-coupled receptors (S1PR1-5), which further regulates the intracellular signal pathways involved in diverse cellular functions. It has been indicated that the cellular kinases PKC, PKA, and ERK1/2 are associated with SPHK1 activation [24, 27, 28]. However, the kinase for SPHK1 phosphorylation remains elusive.

PKR and SPHK1 are both activated by stress signals, and mutually antagonize pro-apoptotic and pro-survival cellular responses, implying that there may be reciprocal links between SPHK1 and PKR during stress response. Using an established ribotoxic stress response (RSR)-mediated cytotoxic model and other stress signals, we investigated the “cross-talk” between two crucial protein kinases: SPHK1 and PKR. PKR is able to phosphorylate SPHK1 via direct interaction, and enhances the downstream pro-survival S1PR1/MAPKs/IKKα pathway. However, phosphorylated SPHK1 preferentially binds to latent PKR, and negates PKR further activation during stress response. This attenuates the IRE1α-dependent endoplasmic reticulum (ER) stress process, and consequently protects cells from stress-induced cell death or apoptosis. Our results confirm the mutual interplay between the two kinases—especially with regard to activation and negative feedback—during cellular stress response. They also reveal that cell fate is probably determined by newly built homeostasis during stress stimuli via self-balance in the phosphorylation levels of SPHK1 and PKR.

## Results

### Multiple stress factors increase the phosphorylation of PKR and SPHK1

**Fig. 1:**
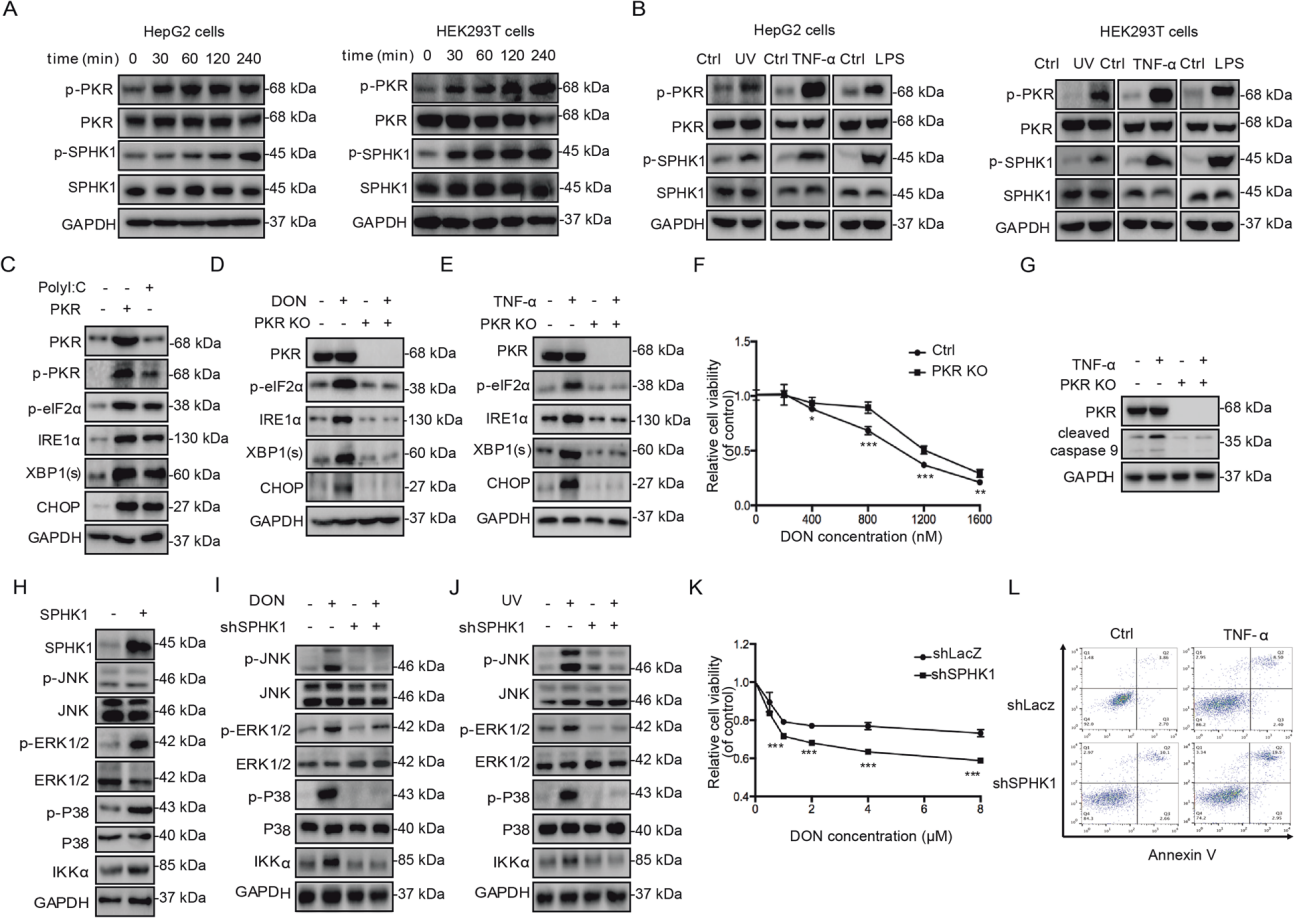
Multiple stress factors increase the phosphorylation of PKR and SPHK1. **a** The levels of phosphorylated PKR and SPHK1 were evaluated following treatment with 2 μM or 400 nM DON for the indicated time periods in HepG2 and HEK293T cells. The cells were then harvested and subjected to western blotting analysis. The dosages of DON administrated in both cells are chosen according to IC50 measurements by CCK-8 assay as shown in Supplementary Fig. 1. **b** The levels of phosphorylated PKR and SPHK1 were evaluated following irradiation with UVC for 10 min, treatment with 10 ng/mL TNF-α, or treatment with 1 μg/mL LPS for 3 h in HepG2 and HEK293T cells. The cells were then harvested and subjected to western blotting analysis. **c** The expression of ER stress-related proteins were evaluated during PKR overexpression and PolyI:C administration. HEK293T cells were transfected with pCDNA3.1–PKR or pCDNA3.1 for 24 h or treated with 10 μg/mL PolyI:C for 3 h. The optimization of PolyI:C concentrations as the positive control is shown in Supplementary Fig. 5. The cells were then harvested and subjected to western blotting analysis. **d**, **e** ER stress-related protein levels during DON or TNF-α exposure were evaluated in PKR knockout cells. HEK293T control and PKR knockout cells at 70% confluence were incubated with 400 nM DON or 10 ng/mL TNF-α for 3 h. The cells were then harvested and subjected to western blotting analysis. **f** Cytotoxicity of DON was evaluated in PKR knockout cells. HEK293T control and PKR knockout cells at 70% confluence were treated with various concentrations of DON for 24 h. Cell viability was then determined with CCK-8 (*n* = 6). **g** Cell apoptosis resulting from TNF-α treatment in PKR knockout cells. HEK293T control and PKR knockout cells were evaluated with 10 ng/mL TNF-α by targeting cleaved caspase 9. The cells were then harvested and subjected to western blotting analysis. **h** The expression levels of IKKα and phosphorylated MAPKs during SPHK1 overexpression. HepG2 cells were transfected with pCDNA3.1–SPHK1 or pCDNA3.1 for 24 h. The cells were then harvested and subjected to western blotting analysis. **i**, **j** The expression levels of DON- and UV-induced IKKα and phosphorylated MAPKs were evaluated in shSPHK1 cells. HepG2 shLacZ and shSPHK1 cells at 70% confluence were incubated with 2 μM DON for 3 h or exposed to UVC irradiation for 10 min. The cells were then harvested and subjected to western blotting analysis. **k** Cytotoxicity of DON in shSPHK1 cells. HepG2 shLacZ and shSPHK1 cells at 70% confluence were treated with various concentrations of DON for 24 h. Cell viability was then determined with CCK-8 (*n* = 6). **l** Cell apoptosis resulting from TNF-α treatment in shSPHK1 cells. HepG2 shLacZ and shSPHK1 cells were evaluated with 10 ng/mL TNF-α by flow cytometry. The data were analyzed using FlowJo software. The results are the means ± SEMs of at least three independent experiments. Statistical significance was defined as *p < 0.05, **p < 0.01, or ***p < 0.001.

Phosphorylated PKR is generally considered a critical stress sensor protein and a biomarker of RSR [29, 30]. SPHK1 is widely accepted as an important responsive kinase of many bioactivators, especially growth and survival factors [21]. It therefore increases SPHK1 activity, as proved by the enhancement of Ser225 phosphorylation [24]. Deoxynivalenol (DON) and UVC irradiation as typical cellular stress inducers, and two canonical stress indicators (LPS and TNF-α) as positive controls, were administrated to HepG2 and HEK293T cells, as representative cells of the major target organs, the liver, and kidney, respectively. Surprisingly, DON exposure significantly increased the phosphorylation of PKR and SPHK1 over a broad time-range, and SPHK1 phosphorylation presented a lag effect compared with phosphorylated PKR in both cell lines (Fig. 1a and Supplementary Fig. 2). We observed similar activation effects in the UVC-irradiated, TNF-α and LPS-treated cells (Fig. 1b). These results suggest that the typical RSR inducers induce the phosphorylation of SPHK1, which reveals a novel underlying stress-sensitive function of SPHK1.

DON and TNF-α were also able to activate IRE1α/XBP1/CHOP signals within the indicated cell lines (Supplementary Fig. 3). This activation refers to the ER stress response, another key apoptotic signaling pathway, which is also regarded as a critical process responsible for cytotoxicity in stress response [31]. This co-occurrence of RSR and the ER response during stress stimulation implies a causative relationship. Therefore, we manipulated PKR expression to investigate the potential relationship between these pathways. Either administration of PolyI:C, the canonical stimulus for PKR phosphorylation or overexpression of PKR significantly increased IRE1/XBP1/CHOP expression (Fig. 1c). Meanwhile, PKR knockdown and knockout cell lines were constructed based on shRNA-expressing H1 retroviral system and CRISPR/Cpf1 system (Supplementary Fig. 4) [32]. IRE1α/XBP1/CHOP expression was significantly suppressed in comparison with that in the wild-type cells, regardless of the basal or induced conditions (Fig. 1d, e and Supplementary Fig. 6). This indicates that the activation of IRE1α signals probably depends on the level of PKR phosphorylation. In agreement with the previous studies—the loss of PKR function decreases antiviral activity and attenuates the cellular pro-apoptotic responses caused by several cytokines and growth factors [33]—PKR deficiency in the HEK293T cells did protect them from death and apoptosis when exposed to DON or TNF-a (Fig. 1f, g and Supplementary Fig. 7). Collectively, the data indicate that PKR serves as a key regulatory factor that transduces the stress signals from RSR to ER stress, and ultimately leads to cell death and apoptosis.

It is generally accepted that the activation of NF-κB is responsible for apoptosis resistance, and the activation of MAPKs promotes cell survival [34, 35]. To clarify the physiological function of SPHK1 during stress response, we determined the activation levels of ERK1/2, p38, JNKs, and IKKα during stress response in shSPHK1 cells or in the control cells. Overexpression of SPHK1 effectively elevates the levels of IKKα and the phosphorylated MAPKs. However, we found that this activation was markedly abrogated in shSPHK1 cells (Fig. 1h–j). Also, the inhibition of IKKα and phosphorylated MAPKs by specific inhibitors significantly increased the cytotoxicity of DON in the HepG2 cells, suggesting that the activation of these pro-survival kinases essentially antagonizes cell death during stress response (Supplementary Fig. 8). Meanwhile, SPHK1 knockdown apparently aggravated cell death or apoptosis during exposure to DON and TNF-α, respectively (Fig. 1k, l), revealing a pro-survival funciton of SPHK1 in stress response. In general, the activation of PKR and SPHK1 is considered as the common mechanism in the cellular response to various external stimuli for cell apoptosis and survival.

### SPHK1 is directly phosphorylated by activated PKR

**Fig. 2:**
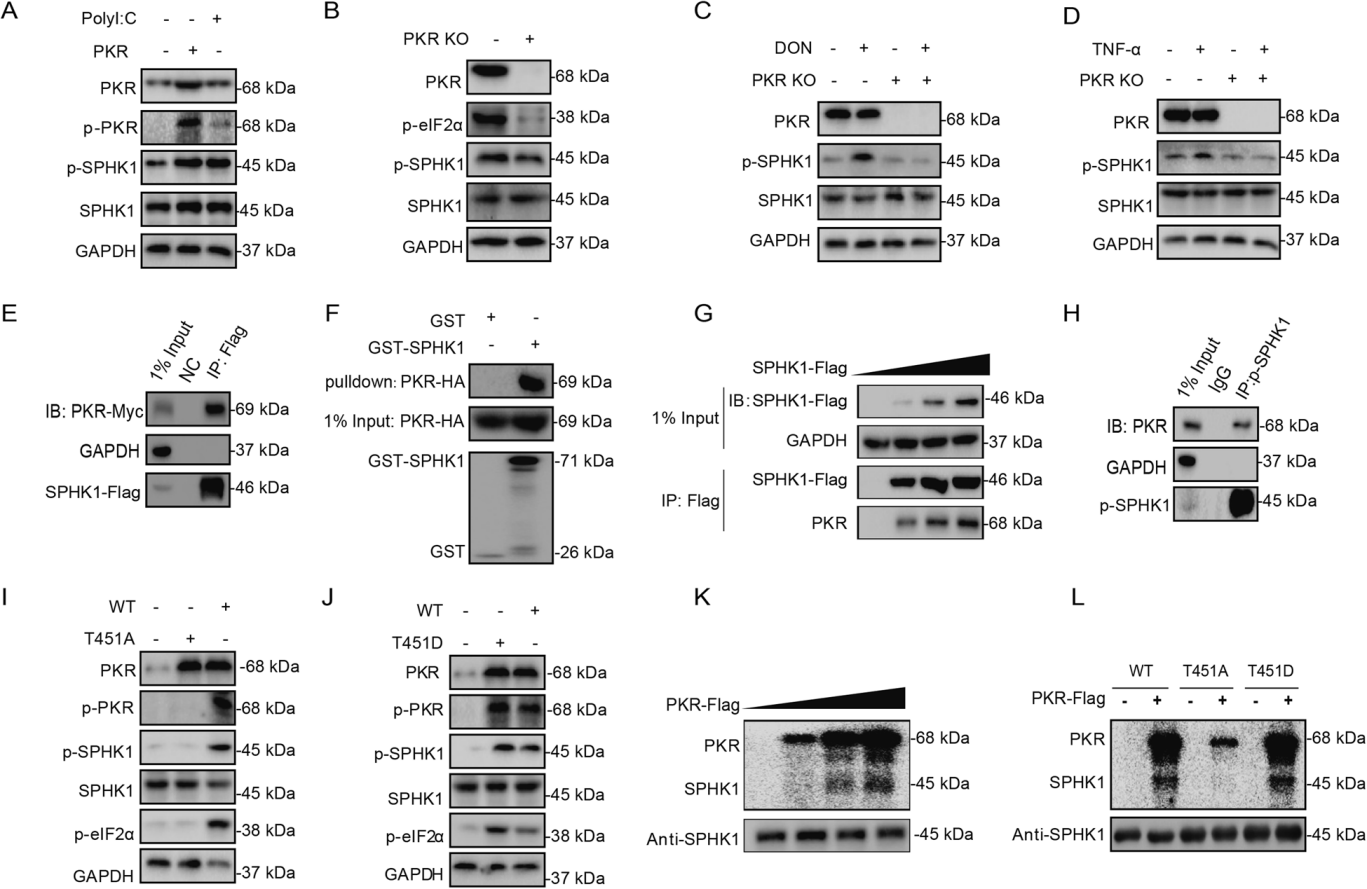
SPHK1 is directly phosphorylated by activated PKR. **a** The phosphorylation levels of SPHK1 were evaluated during PKR overexpression and PolyI:C administration. HEK293T cells were transfected with pCDNA3.1–PKR or pCDNA3.1 for 24 h and then treated with 10 μg/mL PolyI:C for 3 h. The cells were then harvested and subjected to western blotting analysis. **b** The levels of SPHK1 phosphorylation were evaluated in PKR knockout cells. HEK293T control and PKR knockout cells at 90% confluence were harvested and subjected to western blotting analysis. **c**, **d** The expression levels of phosphorylated SPHK1 were evaluated following treatment with DON or TNF‐α in PKR knockout cells. HEK293T control and PKR knockout cells at 70% confluence were incubated with 400 nM DON or 10 ng/mL TNF-α for 3 h. The cells were then harvested and subjected to western blotting analysis. **e** Co-IP analysis of the interaction between SPHK1 and PKR. HEK293T cells were co-transfected with SPHK1-Flag and PKR-Myc for 24 h. The cell lysates were subjected to immunoprecipitation with the indicated antibodies, and visualized by western blotting. pCDNA3.1 transfection was used as a negative control. **f** GST pulldown analysis of the interaction between SPHK1 and PKR. Purified GST and GST–SPHK1 protein bound to agarose beads were added to the lysate of 293T cells overexpressing PKR-HA. GST protein was used as a negative control. **g** Whole extracts from HEK293T cells transfected with 0, 1, 2, and 3 μg of DNA encoding SPHK1-Flag protein were processed for immunoprecipitation with antibodies against Flag or PKR. **h** Immunoprecipitation analysis of the endogenous interaction between phosphorylated SPHK1 and PKR. Cell lysates of HEK293T cells were subjected to immunoprecipitation with the indicated antibodies, and visualized by western blotting. **i**, **j** The effect of the PKR Thr451 amino acid position on SPHK1 phosphorylation. HEK293T cells at 70% confluence were transfected with wild-type PKR, the PKR^451A^ mutant, or the PKR^451D^ mutant for 24 h. The cells were then harvested and subjected to western blotting analysis. **k**, **l** In vitro kinase assay to determine the activity of SPHK1 using γ-^32^P ATP labeling. Cell lysates from transfected wild-type PKR or its mutants were purified using Flag (M2) magnetic beads, and mixed with equal aliquots of SPHK1. The kinase reactions were conducted in the presence of 0.5 μCi γ-^32^P ATP, and the products were subjected to sodium dodecyl sulfate–polyacrylamide gel electrophoresis (SDS-PAGE). The ^32^P-incorporated products were then transferred to a phosphor screen and developed using a PerkinElmer scanner. The reactions of PKR and its substrate eIF2α were shown as positive controls in Supplementary Fig.10.

The phosphorylation of SPHK1 and PKR was simultaneously increased following cellular stress stimuli. However, whether these two kinases are activated in a chronological order remains unclear. Surprisingly, we found that the activation of phosphorylated SPHK1 was increased markedly in the PKR-overexpressed or PolyI:C-treated cells, but blocked in the PKR knockout or knockdown cells in basal or induced conditions (Fig. 2a–d and Supplementary Fig. 9). This suggests that PKR is critical in the phosphorylation of SPHK1 at Ser225. Next, we determined whether SPHK1 is phosphorylated by PKR through a direct protein–protein interaction. Using co-immunoprecipitation (Co-IP) and glutathione-S-transferase (GST) pulldown assays, we confirmed the interaction between two kinases (Fig. 2e, f). The increasing of exogenous SPHK1 is correlated to the precipitated amount of endogenous PKR in a dosage-dependent manner (Fig. 2g). In further, the endogenous phosphorylated SPHK1 co-immunoprecipitated PKR (Fig. 2h), suggesting the direct interaction between two proteins. Given that PKR comprising phosphorylated Thr451 is the main activation form of the kinase, we substituted Thr451 in the PKR with aspartic acid (T451D) or alanine (T451A) to mimic the retention or loss of its kinase activity, respectively. Compared to the wild-type PKR, the PKR^451D^ mutant exhibited higher SPHK1 phosphorylation efficiency, and the PKR^451A^ mutant attenuated this capability, which verified the critical importance of the Thr451 phosphorylation of PKR in the SPHK1 phosphorylation of Ser225 (Fig. 2i, j). Next, using in vitro experimental labeling with γ-^32^P ATP (Fig. 2k, l), the purified His-SPHK1 activity was increased in line with the amount of PKR, and apparent phosphorylation observed only when SPHK1 was co-incubated with wild-type PKR or the PKR^451D^ mutant. Overall, we demonstrated that SPHK1 is a previously unrecognized substrate of PKR.

### Phosphorylated SPHK1 protects cells from stress-induced cytotoxicity by activating the SPHK1/S1PR1/MAPKs/IKKα axis

**Fig. 3:**
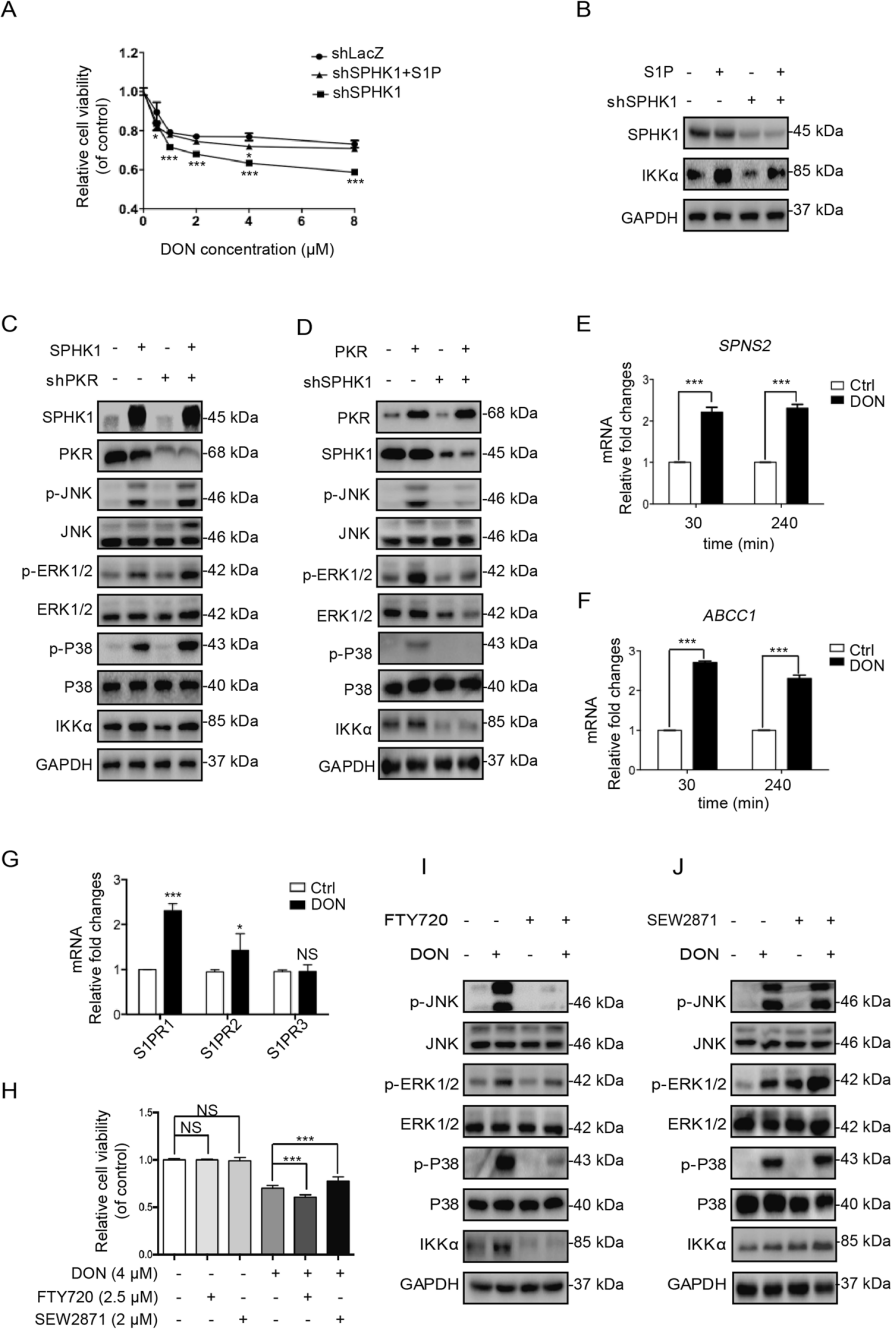
Phosphorylated SPHK1 protects cells from stress-induced cytotoxicity by activating the SPHK1/S1PR1/MAPKs/IKKα axis. **a** The cytotoxicity of DON following S1P exposure in shSPHK1 cells. HepG2 shLacZ or shSPHK1 cells at 70% confluence were treated with various concentrations of DON or with 5 μM S1P added for 24 h. Cell viability was then evaluated with CCK-8 (*n* = 6). **b** The effect of S1P on IKKα protein expression in shSPHK1 cells. HepG2 shLacZ or shSPHK1 cells at 70% confluence were treated with 5 μM S1P for 3 h. IKKα protein expression was then evaluated by western blotting analysis. **c** The effect of SPHK1 overexpression on the expression of IKKα and phosphorylated MAPKs in shPKR cells. HEK293T shLacZ or shPKR cells at 70% confluence were transfected with pCDNA3.1–SPHK1 or pCDNA3.1 for 24 h. The cells were then harvested and subjected to western blotting analysis. **d** The effect of PKR overexpression on the expression of IKKα and phosphorylated MAPKs in shSPHK1 cells. HepG2 shLacZ or shSPHK1 cells at 70% confluence were transfected with pCDNA3.1–PKR or pCDNA3.1 for 24 h. The cells were then harvested and subjected to western blotting analysis. **e**, **f** Fold changes in the mRNA levels of *SPNS2* and *ABCC1* were evaluated following treatment with 2 μM DON for 30 min or 240 min in HepG2 cells. The *SPNS2* and *ABCC1* mRNA levels were normalized to those of GAPDH (*n* = 6). **g** Fold changes in the mRNA levels of *S1PRs* were evaluated following treatment with 2 μM DON for 3 h. Then mRNA levels of *S1PR1*, *S1PR2*, and *S1PR3* were determined by qRT-PCR, and normalized to those of GAPDH (*n* = 6). **h** The effect of S1PR1 on the cytotoxicity of DON. HepG2 cells were pre-incubated with S1PR1 antagonist (2.5 μM FTY720) and agonist (2 μM SEW2871) for 1 h. The cells were then incubated with 2 μM DON for 24 h. Cell viability was evaluated with CCK-8 (*n* = 6). **i**, **j** The effect of S1PR1 antagonist FTY720 and agonist SEW2871 on the DON-induced expression of IKKα and phosphorylated MAPKs. HepG2 cells at 70% confluence were pre-incubated with 1 μM FTY720 or 1 μM SEW2871 for 1 h, and incubated with 2 μM DON for 3 h, then subjected to western blotting analysis. The results are the means ± SEMs of at least three independent experiments. Statistical significance was defined as *p < 0.05, **p < 0.01, or ***p < 0.001.

S1P is the main product of SPHK1 phosphorylating the substrate sphingosine. We observed that S1P accretion provided a significant replenishment of cell survival in SPHK1 knockdown cells, as well as IKKα expression (Fig. 3a, b). In contrast, the overexpression of SPHK1 apparently activated IKKα and phosphorylated MAPK signals in PKR knockdown cells. Importantly, PKR overexpression, which induced such activation, was blocked by SPHK1 deficiency. Taken together, these results suggest that downstream pathways are not directly driven by PKR activation, but by SPHK1 phosphorylation (Fig. 3c, d). We evaluated the expression of the three main S1PR isoforms (S1PR1-3) and the S1P transporters, SPNS2, and ABCC1, following DON treatment. S1PR1 and both transporters were significantly upregulated (Fig. 3e–g). The inhibition of S1PR1 by specific inhibitor FTY720 significantly increased the cytotoxicity of DON, whereas the agonist SEW2871 had the opposite effect (Fig. 3h), suggesting that SPHK1/S1P/S1PR1 axis plays an important role in the pro-survival signaling pathway during stress response. The activation or inactivation of IKKα and phosphorylated MAPKs can apparently be manipulated by the addition of S1PR1 inhibitor FTY720 or agonist SEW2871 in HepG2 cells (Fig. 3i, j), confirming that MAPK and IKKα are the downstream effector kinases of the SPHK1/S1P/S1PR1 axis. Taken together, our data indicate that the activation of SPHK1 promotes the S1PR1/MAPKs/IKKα pathway in stress-stimulated HepG2 cells, rather than the direct involvement of phosphorylated PKR.

### Phosphorylated SPHK1 affects PKR activation and ER stress signal pathway

**Fig. 4:**
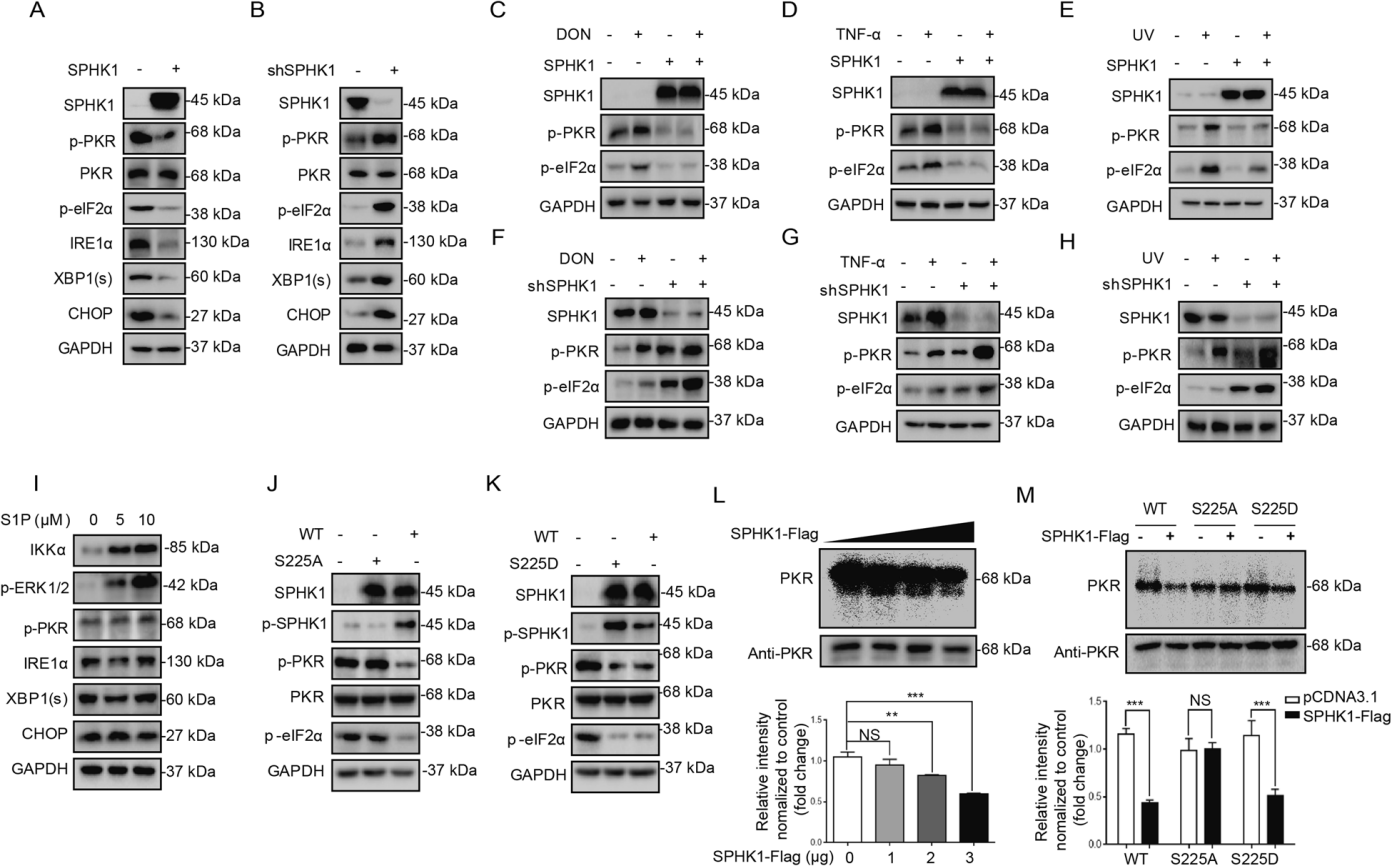
Phosphorylated SPHK1 affects PKR activation and ER stress signal pathway. **a** The expression levels of phosphorylated PKR and ER stress-related protein were evaluated following SPHK1 overexpression. HepG2 cells were transfected with pCDNA3.1-SPHK1 or pCDNA3.1 for 24 h. The cells were then harvested and subjected to western blotting analysis. The exposure time for SPHK1 was 0.3 s, and the exposure time for phosphorylated PKR, phosphorylated eIF2α, IRE1α, XBP1(s), and CHOP was 6 s (the exposure time for these endogenous proteins was 2 s). **b** The expression levels of phosphorylated PKR and ER stress-related protein were evaluated in shSPHK1 cells. HepG2 shLacZ or shSPHK1 cells at 90% confluence were harvested and subjected to western blotting analysis. **c–e** DON, TNF‐α, and UV-induced phosphorylated PKR and eIF2α activation were evaluated following SPHK1 overexpression. HepG2 cells at 70% confluence were transfected with pCDNA3.1–SPHK1 or pCDNA3.1 for 24 h. The cells were then incubated with 2 μM DON or 10 ng/mL TNF-α for 3 h, or exposed to UVC radiation for 10 min. The cells were then harvested and subjected to western blotting analysis. **f–h** DON, TNF-α, and UV-induced phosphorylated PKR and eIF2α activation in shSPHK1 cells. HepG2 shLacZ or shSPHK1 cells at 70% confluence were incubated with 2 μM DON or 10 ng/mL TNF-α for 3 h, or exposed to UVC irradiation for 10 min. The cells were then harvested and subjected to western blotting analysis. (i) The expression levels of phosphorylated PKR and ER stress-related protein were evaluated following S1P exposure. HepG2 cells at 70% confluence were incubated with 5 or 10 μM S1P for 3 h. The expression levels of the target proteins were then determined by western blotting analysis. **j**, **k** The effect of the position of amino acid Ser225 in SPHK1 on PKR phosphorylation. HEK293T cells at 70% confluence were transfected with wild-type SPHK1, the SPHK1^225A^ mutant, or the SPHK1^225D^ mutant for 24 h. The cells were then harvested and subjected to western blotting analysis. **l**, **m** In vitro kinase assay of the activity of PKR using γ-^32^P ATP labeling. Cell lysates from transfected Flag-tagged SPHK1 or its mutants were purified using Flag (M2) magnetic beads, and were mixed with equal aliquots of PKR. The kinase ^32^P labeling reactions were conducted in the presence of 0.5 μCi γ-^32^P ATP, and the products were subjected to sodium dodecyl sulfate–polyacrylamide gel electrophoresis (SDS-PAGE). The ^32^P-incorporated proteins were then transferred to a phosphor screen and developed using a PerkinElmer scanner. The results are the means ± SEMs of at least three independent experiments. Statistical significance was defined as *p < 0.05, **p < 0.01, or ***p < 0.001.

We confirmed that phosphorylated PKR activates SPHK1 kinase activity, but it remained necessary to determine whether there has mutual correlation or any reciprocal effect between these two kinases in stressed cells. The overexpression of SPHK1 did not reduce the level of PKR protein, but did suppress the phosphorylation of PKR and its substrate eIF2α under both basal and stress-induced conditions, which further led to an decrease in IRE1α-dependent ER stress signals (Fig. 4a, c–e). In contrast, SPHK1 knockdown increased the phosphorylation of PKR, and thus activated its downstream signals (Fig. 4b, f–h). However, exposure to S1P had no apparent suppression effect on the phosphorylation of PKR and downstream regulators during ER stress (Fig. 4i), suggesting a noncanonical role of SPHK1-mediated suppression of PKR phosphorylation is independent of S1P.

To address the role of the core activation site of SPHK1 in suppressing PKR activity, we overexpressed the genes encoding either wild-type SPHK1 or the mutants SPHK1^225A^ and SPHK1^225D^. SPHK1^225D^ overexpression significantly reduced the phosphorylation level of PKR and its substrate eIF2α, as did the wild-type SPHK1, whereas the phosphorylation-deficient SPHK1^225A^ did not (Fig. 4j, k). In vitro experimental labeling with γ‐^32^P ATP, and an ADP-Glo kinase assay further confirmed that SPHK1 suppresses PKR activity (Fig. 4l and Supplementary Fig. 11). The results suggested that the phosphorylation of SPHK1 at Ser225 is crucial in suppressing PKR activity (Fig. 4m).

### Phosphorylated SPHK1 blocks PKR homodimerization by binding to its catalytic domain

**Fig. 5:**
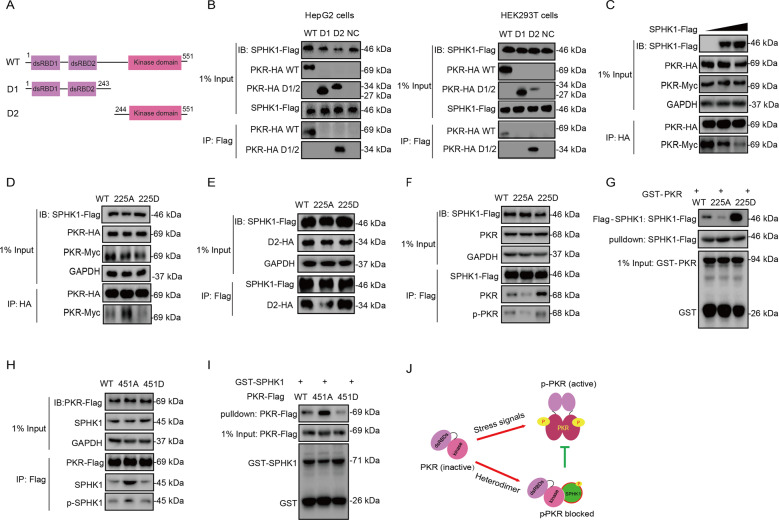
Phosphorylated SPHK1 blocks PKR homodimerization by binding to its catalytic domain. **a** Two HA-tagged PKR deletion mutants were constructed as depicted in the diagram. **b** Identification of the PKR domain responsible for interacting with SPHK1. HepG2 and HEK293T cells were transfected with SPHK1-Flag and the two PKR-HA deletion mutants. The cell lysates were immunoprecipitated with an anti-Flag antibody, and the precipitates and whole-cell lysates were then analyzed by western blotting. **c** The effect of the position of amino acid Ser225 in SPHK1 on SPHK1–PKR interaction. Wild-type/225A/225D Flag-tagged SPHK1 was transfected into HEK293T cells and processed for immunoprecipitation with the indicated antibodies. **d** PKR dimerization was evaluated following SPHK1 overexpression. SPHK1-Flag (0, 1.5 or 3 μg) and an equivalent amount of HA/Myc-tagged PKR were transfected into HEK293T cells and processed for immunoprecipitation. The precipitates and whole-cell lysates were then analyzed by western blotting. **e** The effect of the position of amino acid S225 in SPHK1 on PKR dimerization. Wild-type/225A/225D Flag-tagged SPHK1 and an equivalent amount of HA/Myc-tagged PKR were transfected into HEK293T cells and processed for immunoprecipitation. The precipitates and whole-cell lysates were then analyzed by western blotting. **f** The effect of the position of amino acid Ser225 in SPHK1 on SPHK1–PKR interaction. Wild-type/225A/225D Flag-tagged SPHK1 was transfected into HEK293T cells and processed for immunoprecipitation with an anti-Flag antibody. The cell lysates were then purified and co-incubated with GST-PKR for GST pulldown analysis. **g**, **h** The effect of the position of amino acid Thr451 in PKR on PKR–SPHK1 interaction. Wild-type/451A/451D Flag-tagged PKR was transfected into HEK293T cells and processed for immunoprecipitation with the indicated antibodies, or purified and co-incubated with GST-SPHK1 for GST pulldown analysis. **i** A sketch depicting the mechanism by which phosphorylated SPHK1 binds to the catalytic domain of PKR and blocks PKR autophosphorylation.

Although it has been confirmed that the phosphorylation of SPHK1 inhibits PKR activity, the underlying mechanism remains unclear. To determine the domain responsible for the interaction between PKR and SPHK1, we co-transfected the two truncated mutants of PKR with wild-type SPHK1 in indicated cells (Fig. 5a). Co-IP assay suggested that the catalytic domain of PKR is necessary for interaction with SPHK1 (Fig. 5b). PKR undergoes autophosphorylation through homodimerization, an intermolecular reaction requiring physical interaction between two PKR molecules [36]. As the control for PKR activation, PACT binds to the PKR dsRBD and mediates its activation through a protein interaction mechanism [12]. The interaction between PACT and PKR was not affected by the overexpression of SPHK1 (Supplementary Fig. 12). However, the homodimerization of PKR decreased markedly, as evidenced that much less Myc-tagged PKR was co-immunoprecipitated with HA-tagged PKR (Fig. 5c, d), suggesting that phosphorylated SPHK1 acts as a negative regulator during the formation of the PKR homodimer. Interestingly, as of the wild-type PKR, the kinase domain prefers to interact with phosphorylated SPHK1 (Fig. 5e). To further clarify whether Ser225 of SPHK1 were related to the interaction, we expressed the SPHK1 wild-type and mutants in HEK293T cells to conduct Co-IP and GST pulldown assays. Co-IP suggested that overexpressed SPHK1^225D^ had a stronger interaction with endogenous PKR and phosphorylated PKR than that of SPHK1 wild-type or SPHK1^225A^ (Fig. 5f). GST pulldown assay confirmed SPHK1^225D^ interacts more stronger with PKR in vitro (Fig. 5g). Using similar methods, we assessed the difference in interaction between PKR and its T451 mutants by immunoprecipitating them with endogenous SPHK1 and phosphorylated SPHK1. Co-IP and GST pulldown revealed that unphosphorylated PKR^451A^ has a higher affinity for endogenous or purified SPHK1 than the wild-type PKR or the PKR^451D^ mutant, whereas exhibits the difference to a less extent with endogenous phosphorylated SPHK1(Fig. 5h, i). These results suggest that the activated SPHK1 that had undergone phosphorylation at S225 preferentially interacted with unphosphorylated PKR in HEK293T cells. A sketch map illustrating the mechanism is shown in Fig. 5j.

### PKR is required to phosphorylate SPHK1 in the nucleus and mediate phosphorylated SPHK1 translocation

**Fig. 6:**
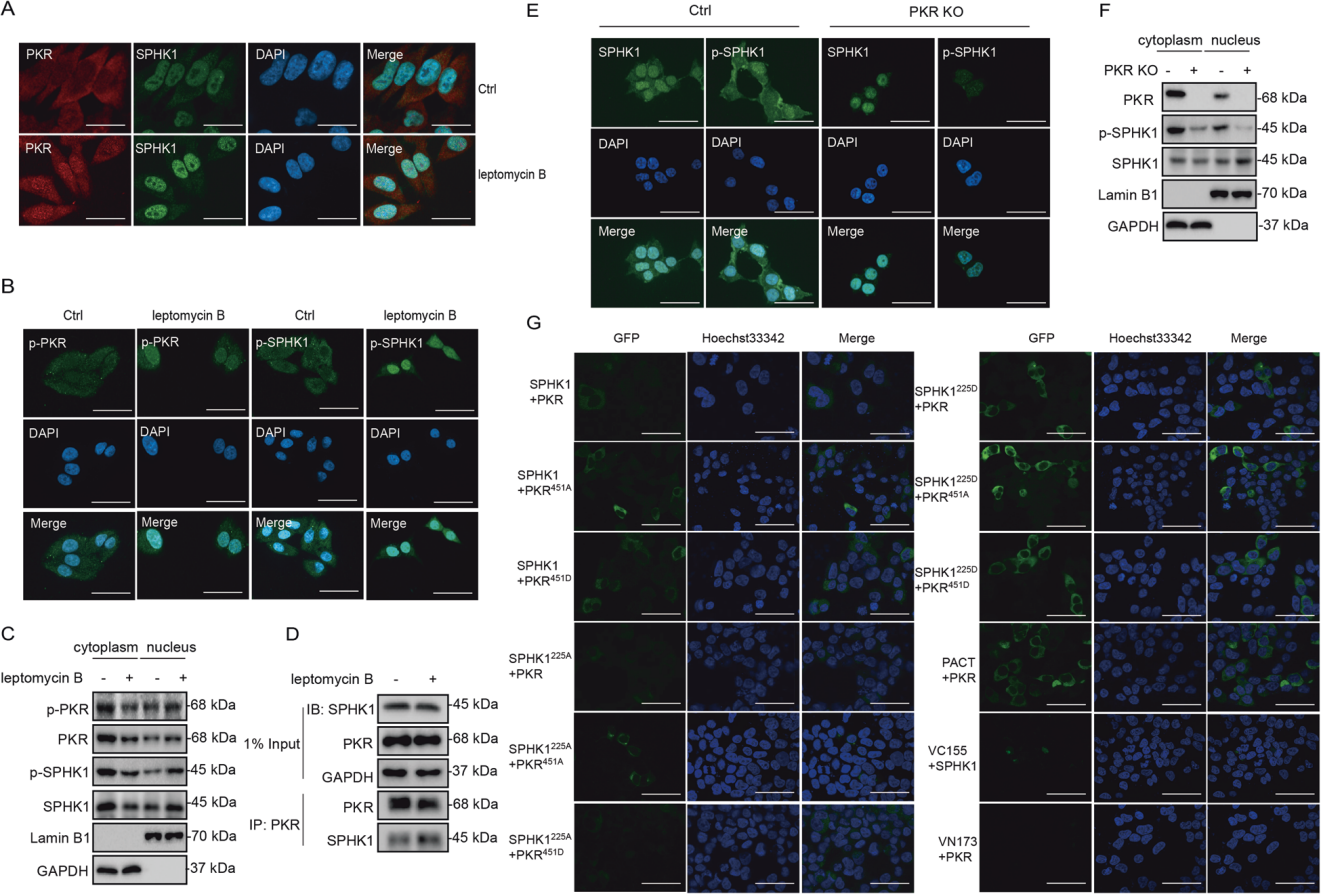
PKR is required to phosphorylate SPHK1 in the nucleus and mediate phosphorylated SPHK1 translocation. **a** The localization of SPHK1 and PKR was evaluated with leptomycin B treatment by an immunofluorescence assay. HEK293T cells were treated with 2 ng/mL leptomycin B for 2 h, and then the cells were fixed and stained with anti-PKR antibodies (red), anti-SPHK1 antibodies (green) and DAPI (blue) (scale bar = 20 μm). **b** The localization of phosphorylated SPHK1 and PKR was evaluated with leptomycin B treatment by an immunofluorescence assay. HEK293T cells were treated with 2 ng/mL leptomycin B for 2 h, and then the cells were fixed and stained with anti-p-PKR antibodies, anti-p-SPHK1 antibodies (green) and DAPI (blue) (scale bar = 50 μm). **c** The protein amounts of SPHK1 and PKR and their phosphorylation levels were evaluated in cytoplasmic and nuclear fractions of HEK293T cells following leptomycin B treatment. GAPDH and Lamin B1 were analyzed as loading controls for the cytoplasmic and nuclear fractions, respectively. **d** Immunoprecipitation analysis of the endogenous interaction between SPHK1 and PKR with leptomycin B treatment. HEK293T cells were treated with 2 ng/mL leptomycin B for 2 h, and then the cell lysates were subjected to immunoprecipitation with the indicated antibodies, and visualized by western blotting. **e** The localization of phosphorylated SPHK1 and SPHK1 was evaluated in PKR knockout cells by an immunofluorescence assay. HEK293T control and PKR knockout cells were fixed and stained with anti-SPHK1 antibodies, anti-p-SPHK1 antibodies (green) and DAPI (blue) (scale bar = 50 μm). **f** The levels of phosphorylated SPHK1 and SPHK1 were evaluated in cytoplasmic and nuclear fractions of PKR knockout cells. GAPDH and Lamin B1 were analyzed as loading controls for the cytoplasmic and nuclear fractions, respectively. **g** The co-localization of SPHK1 with PKR was evaluated by BiFC. PKR-VN173 and SPHK1-VC155 constructs and the mutants were transfected into HEK293T cells, then stained with Hoechst 33342. The figures show representative fluorescent images of the indicated proteins (scale bar = 50 μm).

Previous studies have shown that SPHK1 shuttles between the cytoplasm and the nucleus [37], and SPHK1 translocates to the plasma membrane after phosphorylated at Ser225 [24]. Interestingly, PKR shuttles between the nucleus and the cytoplasm mediated by a CRM1-dependent pathway [38]. In addition, PKR with N-terminal deletion, which removes the autoinhibitory domain of PKR, alters its cytoplasmic predominance to be nuclear enriched. We hypothesized that nuclear PKR occupies a higher phosphorylation state and then possibly mediates the phosphorylation and translocation of SPHK1. Immunofluorescence for phosphorylated PKR suggested that phosphorylation state affects PKR subcellular localization, given that phosphorylated PKR has more apparent nuclear localization than PKR (Fig. 6a, b, the ctrl panels). Both PKR and SPHK1 were apparently enriched in the nucleus when the cells treated with leptomycin B, the specific CRM1 inhibitor which blocks protein exported out of the nucleus (Fig. 6a). The phosphorylated SPHK1 localized differently with SPHK1, mainly cytoplasmic rather than nuclear, was also strongly enriched in the nucleus after leptomycin B treatment (Fig. 6b). These data suggested that both PKR and SPHK1 shuttle between the cytoplasm and the nucleus in HEK293 cells. However, the compartmentation of these two phosphorylated proteins are partially separated—phosphorylated PKR was ubiquitous cellular localized but phosphorylated SPHK1 mainly located in the cytoplasm (Fig. 6b, c). The interaction between PKR and SPHK1 was enhanced in leptomycin B treated cells (Fig. 6d), suggesting that PKR is able to interact with SPHK1 in the nucleus and possibly phosphorylates SPHK1. In PKR knockout cells, SPHK1 expression was not affected, but both SPHK1 and phosphorylated SPHK1 in the cytoplasm was reduced (Fig. 6e, f). Compared with the control cells, neither DON nor TNF-a effectively stimulate SPHK1 translocation in PKR knockout cell line (Supplementary Fig. 13). These results suggested that SPHK1 phosphorylation in the nucleus and further translocation to the cytoplasm require the presence of PKR.

A bimolecular fluorescence complementation (BiFC) assay was conducted to assess the interaction intensity between SPHK1 and PKR mutant combinations. The BiFC analysis revealed complemented fluorescence in the HEK293T cells overexpressing PKR and SPHK1, again suggesting that these two proteins interact with each other. The stronger fluorescence in the cells transfected with SPHK1^225D^ complemented with PKR and PKR mutants than that with SPHK1^225A^ (Fig. 6g). Similarly, the amounts of immunoprecipitated SPHK1^225D^ by PKR and its mutants were much more than that of precipitated SPHK1^225A^ (Supplementary Fig. 14b). Both experiments suggest that phosphorylation activated SPHK1 efficiently binds to PKR, and preferentially interacts with unphosphorylated PKR (mimicked by PKR^451A^) in the cytoplasm.

### The homeostasis of SPHK1 and PKR in cells: SPHK1—a previously unrecognized substrate of phosphorylated PKR—provides negative feedback to PKR activation

**Fig. 7:**
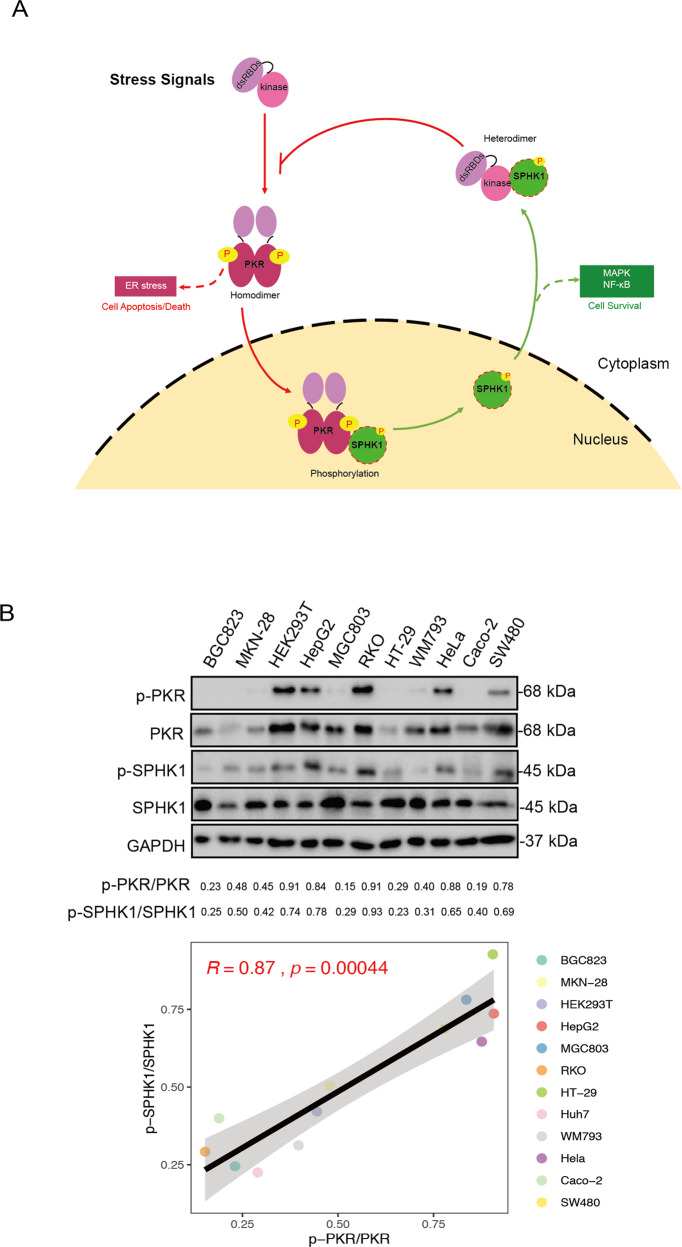
The homeostasis of SPHK1 and PKR in cells: SPHK1—a previously unrecognized substrate of phosphorylated PKR—provides negative feedback to PKR activation. **a** A model depicting the proposed mechanism for the homeostasis of SPHK1 and PKR in cells. **b** The expression levels of phosphorylated PKR and SPHK1 were evaluated in several types of cells. Cells at 90% confluence were harvested and analyzed by western blotting with the indicated antibodies. The phosphorylation ratios of PKR and SPHK1 were noted in the bottom of the blots. Linear correlation between variables was tested by calculating the Pearson’s correlation coefficient.

Our study provides evidence of “cross-talk” between two important stress-related kinases: SPHK1 and PKR. Mechanistically, a portion of activated PKR interacts and phosphorylates SPHK1 in the nucleus and thus mediate SPHK1 export to the cytoplasm, which further binds to the catalytic domain of PKR in the cytoplasm. This blocks the oligomerization of PKR and prevents its autophosphorylation, thereby attenuates further PKR activation and consequently prevents cell death or apoptosis (Fig. 7a). Several researchers have reported that SPHK1 is overexpressed in various types of cancer, whereas a higher PKR content results in a more favorable prognosis [39, 40]. What distinguishes the present study from those mentioned above revealed a novel mechanism—whereby there is a phosphorylation balance between active SPHK1 and PKR. Thus, we examined the levels of phosphorylated SPHK1 and PKR within several cell lines (Fig. 7b). As expected, the phosphorylated level of PKR was found to be significantly correlated with the phosphorylation level of SPHK1 (*R* = 0.87, *p* = 0.00044).

## Discussion

PKR, well characterized as a crucial kinase in response to stress signals, is activated through autophosphorylation, and subsequently activates its substrate eIF2α, mediating the inhibition of translation [41]. Besides the PKR–eIF2α axis, various stress stimuli provoke the ER stress signal, which is another crucial pro-apoptotic pathway [42]. Although few studies suggest that PKR acts as an ER stress-regulated kinase, since the identification of PERK, the role of PKR in ER stress remains unclear [43]. As a typical RSR inducer, DON has been proven to activate multiple intracellular signals, including PKR and ER stress pathways [29, 44]. According to our data, the activation of PKR is the prerequisite for the downstream induction of the IRE1α/XBP1(s)/CHOP-dependent ER stress signals. Taken the pro-apoptotic function mediated by PKR, we hold the opinion that the activated PKR is the pro-apoptotic signaling node that transduces the signals from RSR to ER stress response.

Except of its involvement in pro-apoptotic events, PKR participates in cell proliferation and differentiation processes [45]. This raises the question of whether PKR directly affects the antagonized choices for cell fate, cell death, or survival, especially when cells respond to external stress signals. Interestingly, both PKR and SPHK1 have been suggested as upstream activators of signaling cascades for the activation of MAPKs and NF-κB in response to stress stimuli [29, 46, 47]. Besides LPS and TNF‐α, the RSR inducers DON and UVC radiation, apparently activate PKR and SPHK1 with high levels of phosphorylation. This co-stimulated phosphorylation implicates a potential link between the activation of PKR and that of SPHK1, possibly as a general mechanism that functions in the cellular response to external stimuli.

In this study, we found tight regulatory interplay between the two kinases. First, PKR phosphorylates SPHK1 to produce its activated form through direct interaction. Moreover, PKR phosphorylates SPHK1 dependent on its activation, Thr451 phosphorylation. Consequently, phosphorylated SPHK1 significantly promotes the activation of downstream pro-survival pathways, such as the further activations of MAPKs and NF-kB in the cells.

In shSPHK1 or SPHK1 inhibitor-treated cells, the RSR stress stimuli did not promote the greater activation of IKKα or produce higher levels of phosphorylated MAPKs. Instead, in shPKR cells, overexpression of SPHK1 apparently activated these signals. This proved that these downstream pathways are not directly driven by PKR activation, but by SPHK1 phosphorylation. Administrations of DON and TNF-α caused higher rates of cell death or apoptosis in SPHK1-deficient HepG2 cells, indicating that SPHK1 is required for cell survival during cellular stress response. As a self-protection mechanism in response to stress, it is possible that the activation of SPHK1, driven by PKR phosphorylation, promotes the production of S1P, and activates the S1PR1/MAPKs/IKKα pathway in a stepwise manner. Given that widespread PKR-mediated pathways participate in cellular pro-apoptosis events, it is surprising that the simultaneous activation of SPHK1 serves as a pro-survival mechanism to antagonize the anti-proliferative function of PKR.

An interesting implication is that SPHK1 regulates the core machinery of ER stress-mediated lipotoxicity in hepatocytes [48]. Furthermore, we found that phosphorylated SPHK1 suppresses the IRE1α-mediated ER stress pathway, and is correlated with the downregulation of PKR activation. Highly expressed SPHK1 strongly inhibited PKR activity, and consequently suppressed IRE1α-dependent ER stress signals. In vitro purified SPHK1 retained the ability to suppress PKR activity, but that was not true of the dysfunctional mutant SPHK1^225A^, either in vivo or in vitro.

Truncated protein interaction suggests that SPHK1 binds to the kinase domain of PKR directly, and this interaction further blocks PKR homodimerization, as evidenced by the disruption of PKR–PKR interaction revealed by a competitive combination strategy. This interaction-based mechanism implies that phosphorylated SPHK1 efficiently binds to latent PKR, suggesting a feedback mechanism by which phosphorylated SPHK1 regulates the suppression of PKR activity. It is worth noting that both PKR and SPHK1 shuttle between the cytoplasm and the nucleus in the cells, though their main localizations are spatially separated. Our data support the envision of the two kinase interplay as that some stress signals stimulate the activation of PKR in the cytoplasm by autophosphorylation, the phosphorylated PKR is able to shuttle into the nucleus and then probably phosphorylates SPHK1, and subsequently the phosphorylated SPHK1 preferentially translocates to the cytoplasm to bind the latent PKR to prevent the further homodimerization-mediated activation of PKR. This might be the physiological importance of this compartmentation separation for most of phosphorylated PKR and phosphorylated SPHK1 which allows phosphorylated SPHK1 to negate the further PKR activation after translocated to the cytoplasm especially under stress conditions. Having demonstrated the effect of PKR phosphorylation on SPHK1, this negative regulation revealed a “self-protection” mechanism in the process of cell homeostasis that is maintained by balancing the activation of these two sensor kinases. This conclusion was further verified by the positive correlation between phosphorylated PKR and SPHK1 in a variety of cell lines.

In conclusion, the phosphorylation of SPHK1 and PKR is a feature of upstream activation signals during stress response. We discovered a novel role of SPHK1, i.e., as a substrate of PKR, and the phosphorylation of SPHK1 driven by stress-induced PKR activation promotes cellular pro-survival signals. More importantly, activated SPHK1 acts as a negative regulator to maintain the new cellular homeostasis via antagonizing the apoptotic pathways by directly binding to PKR and inhibiting its further autophosphorylation, which physiologically redefines cell fate. For the first time, our study provides evidence that the activation of SPHK1 by stress contributes to a cross-coupling reaction with PKR during cell adaptation. This ultimately provides a new perspective on the upstream signals related to cell death and survival.

## Materials and methods

### Chemical reagents

DON, TNF-α, LPS, PolyI:C, and DMSO were purchased from Sigma Aldrich (St. Louis, MO, USA). FTY720, SEW2871, SB203580, SCH772984, SP600125, and IKK-16 were purchased from Selleck (Shanghai, China). S1P (d18:1) was purchased from Cayman Chemical (Michigan, USA). The cell culture media DMEM, 0.25 % trypsin-EDTA, and puromycin were obtained from Invitrogen (Carlsbad, CA, USA). Hoechst 33342 and 4′,6-diamidino-2-phenylindole (DAPI) were obtained from Beyotime Biotech (Shanghai, China). All other chemicals were analytical grade.

### Antibodies

The antibodies applied in this study are listed in Supplementary Table 1.

### Plasmids

We cloned full-length complementary DNAs (cDNAs) encoding human SPHK1 and PKR into pGEX-4T1/pET28a or pcDNA3.1 vectors for prokaryotic or mammalian cell expression studies. The primers used are listed in Supplementary Table 2. For mammalian cell transfection, the deletion mutants and point mutants of SPHK1 and PKR were prepared by mutagenesis, and verified by sequencing. For the Co-IP assay, Flag, Myc, and HA epitope tags were added to the C-terminal coding ends of the SPHK1 and PKR constructs. For BiFC analysis, VN173(1–172) and VC155(155–238), which are complementary fragments of Venus, were fused to the C-termini of SPHK1 and PKR, respectively.

### Cell culture

HepG2 (ATCC, HB-8065), HEK293T (ATCC, CRL-11268), MCF-7(ATCC, HTB-22), SW480(ATCC, CCL-228), WM793(ATCC, CRL-2806), Caco-2(ATCC, HTB-37), RKO (ATCC, CRL-2577), HT-29 (ATCC, HTB-38) and Hela (ATCC, CCL-2) cell lines were preserved in our laboratory. MGC803, BGC823, MKN-28, Huh7, 7703 cell lines were generous gifted by Prof. Xiaofeng Zhu (Affiliated Tumor Hospital, Sun Yat-sen University). All the cell lines were regularly tested and ensure to be negative for mycoplasma contamination. The cells were cultured in DMEM (high d-glucose; Invitrogen, USA) supplemented with 10% FBS (BI, German) at 37 °C in a CO_2_ incubator (Thermo Fisher, USA).

### Stress stimuli

DON, TNF-α, and LPS were administrated to the HepG2 and HEK293T cells at the indicated concentrations and for the indicated time periods. For the ultraviolet irradiation treatment, we placed the HepG2 and HEK293T cells in phosphate-buffered saline (PBS), and irradiated them with UVC light (254 nm) for 10 min, corresponding to 100 Joules/m^2^ at a concentration of 1 million cells/mL.

### Cytotoxicity assay

The cytotoxic effects of DON were determined in indicated cell lines using a cell counting kit-8 (CCK-8) assay (Sangon Biotech, China). Cells were seeded in a 96-well plate at 5000 per well in the presence of the indicated concentration of DON for 24 h in a cell culture incubator. 0.5 mg/mL CCK-8 solution was added to each well for 2 h at 37 °C. The optical density of each well was determined at a wavelength of 450 nm using a microplate reader (Promega, USA).

### Quantitative reverse-transcription polymerase chain reaction (qRT-PCR)

We grew the HepG2 cells on a six-well plate with or without DON for 3 h, then isolated the RNA using TRIZOL reagents (Invitrogen, USA) according to the protocol described in a previous study. The cDNA was synthesized using a PrimeScript™ RT Reagent Kit according to the manufacturer’s protocol (Takara Biotechnology, China). The qRT-PCR samples were prepared using SYBR Green PCR Master Mix (Promega, USA) and the primers were listed in Supplementary Table 2. The samples were optimized for amplification under the following reaction conditions: denaturization at 95 °C for 10 min; followed by 40 cycles at 95 °C for 15 s, and 60 °C for 1 min. The melting curve of each sample was analyzed after completion of the amplification protocol. We used the housekeeping gene that encodes glyceraldehyde 3-phosphate dehydrogenase (*GAPDH*) for the expression control (Tables 1 and 2).

**Table 1:** The antibodies applied in current study.

Antigen	Species	Clonality	Clone	Catalog	Manufacturer
SPHK1	Rabbit	Monoclonal	D1H1L	#12071	Cell Signal Technology (Beverly, MA, USA)
IRE1α	Rabbit	Polyclonal		AI601	Beyotime Biotech (Shanghai, China)
XBP1(s)	Rabbit	Polyclonal		ab37152	Abcam (London, UK)
Caspase 9	Rabbit	Polyclonal		#9502	Cell Signal Technology (Beverly, MA, USA)
PACT	Mouse	Monoclonal	D-4	sc-377103	Santa Cruz Biotechnology (CA, USA)
p-JNK (phospho-Thr183/Tyr185)	Rabbit	Monoclonal	81E11	#4668	Cell Signal Technology (Beverly, MA, USA)
p-P38 (phospho-Thr180/Tyr182)	Rabbit	Monoclonal	D3F9	#4511	Cell Signal Technology (Beverly, MA, USA)
p-ERK1/2 (phospho-Thr185/202)	Rabbit	Polyclonal		AF1891	Beyotime Biotech (Shanghai, China)
p-PKR (phospho-Thr451)	Rabbit	Polyclonal		ab81303	Abcam (London, UK)
GAPDH	Mouse	Monoclonal	6C5	sc-32233	Santa Cruz Biotechnology (CA, USA)
p-SPHK1 (phospho-Ser225)	Rabbit	Polyclonal		19561-1-AP	Proteintech (Rosemont, USA)
PKR	Rabbit	Polyclonal		AF2125	Beyotime Biotech (Shanghai, China)
IKKα	Rabbit	Polyclonal		AF0198	Beyotime Biotech (Shanghai, China)
JNK	Rabbit	Polyclonal		D120893	Sangon Biotech (Shanghai, China)
P38	Rabbit	Polyclonal		AF1111	Beyotime Biotech (Shanghai, China)
ERK1/2	Rabbit	Polyclonal		D151973	Sangon Biotech (Shanghai, China)
p-eIF2α	Rabbit	Polyclonal		AF1237	Beyotime Biotech (Shanghai, China)
eIF2α	Mouse	Polyclonal		D199693	Sangon Biotech (Shanghai, China)
CHOP	Mouse	Polyclonal		D262889	Sangon Biotech (Shanghai, China)
Flag	Rabbit	Polyclonal		F7425	Sigma Aldrich (St. Louis, MO, USA)
Flag	Mouse	Monoclonal	M2	F3165	Sigma Aldrich (St. Louis, MO, USA)
Myc	Rabbit	Monoclonal	71D10	#2278	Cell Signal Technology (Beverly, MA, USA)
Myc	Mouse	Monoclonal	9B11	#2276	Cell Signal Technology (Beverly, MA, USA)
HA	Rabbit	Monoclonal	C29F4	#3724	Cell Signal Technology (Beverly, MA, USA)
HA	Mouse	Monoclonal	6E2	#2367	Cell Signal Technology (Beverly, MA, USA)
GST	Mouse	Monoclonal	B-14	sc-138 HRP	Santa Cruz Biotechnology (CA, USA)
Goat anti Mouse IgG H&L (Alex Fluor 488)	Mouse			ab150117	Abcam (London, UK)
Goat anti-Rabbit IgG H&L (Alex Fluor 647)	Rabbit			ab150079	Abcam (London, UK)
HRP-linked anti-Mouse IgG	Mouse			#3724	Cell Signal Technology (Beverly, MA, USA)
HRP-linked anti-Rabbit IgG	Rabbit			#7074	Cell Signal Technology (Beverly, MA, USA)

**Table 2:** The primers applied in current study.

Gene	Primer
*SPHK1*^a^	
Sense	CCCAAGCTTATGGATCCAGCGGGCGGCCC
Antisense	CCGCTCGAGATGGATCCAGCGGGCGGCCC
*PKR*^a^	
Sense	CGCGGATCCATGGCTGGTGATCTTTCAG
Antisense	CCCAAGCTTACATGTGTGTCGTTCATTTT
*SPHK1*^b^	
Sense	CCGGAATTCATGGATCCAGCGGGCGGCCC
Antisense	CCGCTCGAGTCAATGGATCCAGCGGGCGGC
*PKR*^b^	
Sense	CGCGCATCCATGGCTGGTGATCTTTCAGCA
Antisense	CCGCTCGAGCTAACATGTGTGTCGTTCATTTT
*D1*^c^	
Sense	CTAGTCTAGAATGGCTGGTGATCTTTCAGC
Antisense	CCCAAGCTTCAAAGATCTTTTTGCCTTCCTTTG
*D2*^c^	
Sense	CTAGTCTAGAATGCGTGTTAAATATAATAACGA
Antisense	CCCAAGCTTACATGTGTGTCGTTCATTTTTCT
*S1PR1*^d^	
Sense	GCCTACACAGCTAACCTGCTCTTG
Antisense	TGGCGATGGCGAGGAGACTG
*S1PR2*^d^	
Sense	CCACCACCTCCTGCCACTCC
Antisense	CACCGTGTTGCCCTCCAGAAAC
*S1PR3*^d^	
Sense	GATCCTCTACGCACGCATCTACTTC
Antisense	ACACGCTCACCACAATCACCAC
*GAPDH*^d^	
Sense	AACGGATTTGGTCGTATTGG
Antisense	GATTTTGGAGGGATCTCGC
*ABCC1*^d^	
Sense	CCGTGTTGGTCTCTGTGTTCCTG
Antisense	AAGTCGGCGGCGTAATTCTTAGC
*SPNS2*^d^	
Sense	GTTACTGGCTGGCTGTGGCTTC
Antisense	GCAACACTCGGACCTGGTTCTTG

^a^Primers used for eukaryotic plasmids construction.

^b^Primers used for prokaryotic plasmids construction.

^c^Primers used for truncated plasmids construction.

^d^Primers used for quantitative RT-PCR.

### Western blotting

The total cell lysates were prepared by using cold radioimmunoprecipitation assay (RIPA) lysis buffer (50 mM Tris–HCl pH 7.8, 150 mM NaCl, 1% Triton X-100 pH 7.8) containing protease and phosphatase inhibitors for 30 min on ice, then centrifuged the mixture for 10 min at 14,000 × *g*. The lysates were subjected to sodium dodecyl sulfate–polyacrylamide gel electrophoresis (SDS-PAGE), and transferred to polyvinylidene fluoride membranes (Millipore, USA). We used an enhanced chemiluminescence (ECL) mix to visualize the proteins in a dark room. For the SPHK1 and PKR overexpression assays, the exposure times for the overexpressed proteins were shorter than that for the endogenous proteins, as described in the figure legends.

### shRNA-mediated gene knockdown

RNA interference was carried out by using a shRNA-expressing H1 retroviral system. The RNA-mediated interference of SPHK1 and PKR was performed in HepG2 or HEK293T cells using a pSUPER.Retro.puro vector (Oligoengine) encoding the shRNA sequence. The target sequences for *SPHK1* and *EIF2AK2* (the gene that encodes PKR) were: 5′-GCAGCTTCCTTGAACCATTAT-3′ and 5′-GAGGCGAGAAACTAGACAAAG-3′, respectively. The knockdown efficiency of the target genes was validated by western blotting.

### CRISPR/Cpf1-mediated *PKR* knockout

PKR knockout cell line was constructed by a CRISPR/Cpf1 system. Small guide RNA (5’AGATAGTACTACTCCCTGCTTCTGACGAA TTTCTACTCTTGTAGATGAGTGTCAGCAGCAGTTAAATAC3’) targeting PKR genome was designed and cloned into PY30 plasmid expressing huAsCpf1 and crRNA guide. The PY30-*PKR*-gRNA was transfected in HEK293 cells with 4 days treatment with 2 μg/mL puromycin, then the cell pools were diluted into 96-well plate to perform the clone selection. In the knockout cell line, the coding sequence of *PKR* was introduced a frameshift and therefore no functional protein was produced, which was confirmed by DNA sequencing and western blotting analysis.

### Apoptosis measurement

We performed an Annexin V-fluorescein isothiocyanate (FITC) staining assay as previously described. The cells were seeded in 6-well plates and exposed to TNF-α as indicated for 24 h. The cells were then trypsinized, washed three times with cold PBS, and stained with Annexin V-FITC for 10 min on ice. Positive cells were detected by flow cytometry.

### Immunofluorescence

We grew HepG2 cells on cell slides inside a 24-well plate for 24 h. The medium was then decanted and the wells were washed three times with cold PBS. The cells were then fixed in 4% paraformaldehyde for 15 min and permeabilized in 0.5% Triton X-100 for 5 min. After washing three times with PBS, the cells were blocked for 1 h at 25 °C in PBS with 5% bovine serum albumin. The primary antibodies were diluted by 1:100 in PBS with 1% bovine serum albumin (antibody dilution buffer) and incubated overnight at 4 °C. After washing three times with PBS, Alexa Fluor 488 anti-rabbit and Alexa Fluor 647 anti-mouse antibodies (Cell Signaling, USA) were added to the antibody dilution buffer at 1:500 and 1:1,000 dilutions, respectively. We then added DAPI to the slides, and incubated them for 1 h at room temperature. After washing the slides five times with PBS, we mounted them using ProLong Gold antifade reagent (Invitrogen, USA). We acquired images using a Two-photon super-resolution point scanning confocal microscope (Nikon, Japan) and selected representative images for each sample.

### Co-immunoprecipitation

We placed the HEK293T cells into 60-mm culture dishes and transfected them with Myc-PKR and Flag-SPHK1 using Lipofectamine 2000 reagent (Invitrogen, USA). After transfection for 24 h, we lysed the cells in NETN buffer (20 mM Tris–HCl pH8.0, 100 mM NaCl, 1 mM EDTA, 0.5% NP-40). The cell extract was used to immunoprecipitate Flag with anti-Flag (M2) magnetic beads, as described, and the beads were then washed six times with NETN buffer. We analyzed the immunoprecipitates by western blotting with anti-Myc and anti-Flag antibodies.

### GST pulldown assay

GST and GST-SPHK1 were expressed in Rosetta bacterial cells using standard procedures, and subsequently incubated overnight with Glutathione Sepharose 4s (GE Healthcare) at 4 °C while agitating the mixture. The beads were then washed and resuspended in RIPA buffer. Each lysate from the HEK293T cells was first mixed with agarose beads conjugated with 30 μg of GST fusion protein, then incubated for 4 h at 4 °C while rotating gently. The beads were washed four times with NETN buffer, and the eluted protein samples were further subjected to western blotting analysis of the indicated proteins.

### Kinase assay

For the γ‐^32^P labeling test, we maintained HEK293T cells transfected with Flag-PKR or SPHK1 in Dulbecco’s modified Eagle’s medium with 10% fetal bovine serum. The cells were harvested and lysed by adding an equal volume of NETN buffer. An aliquot of the total protein was immunoprecipitated using Flag monoclonal antibody in the RIPA buffer at 4 °C for 3 h on a rotating wheel. We washed the beads five times in 500 μL of activity buffer (20 mM Tris–HCl pH 7.5, 50 mM KCl, 2 mM MgCl_2_, 2 mM MnCl_2_, 100 U/mL aprotinin, 0.1 mM phenylmethylsulfonyl fluoride, 5% glycerol). The kinase assay was performed with the PKR still attached to the beads in an activity buffer comprising an equivalent amount of purified His-SPHK1 or GST-eIF2α, 20 μM ATP, and 5 μCi of γ‐^32^P ATP at 30 °C for 30 min. Heat inactivation was carried out at 95 °C for 5 min. The labeled proteins were analyzed by SDS-PAGE on a 10% gel. We then transferred the ^32^P-incorporated proteins to a phosphor screen, and visualized them using a PerkinElmer scanner. For the ADP-Glo kinase assay (Promega, USA), we added 25 μL of ADP-Glo reagent to each kinase reaction vessel, and incubated it at room temperature for 40 min. We added 50 μL of kinase detection reagent to each sample, and incubated the mixture at room temperature for an additional 40 min, after which we evaluated the luminescence of each sample using a GloMax 20/20 luminometer (Promega, USA).

### BiFC analysis

We grew HEK293T cells overnight on cleaned coverslips inside a 24-well plate at 37 °C in a cell culture incubator. The PKR-VN173 and SPHK1-VC155 constructs and the mutants were transfected with Lipofectamine 2000 according to the manufacturer’s instructions. After transfection for 24 h, the nuclear DNA of the living cells was stained with Hoechst 33342. We acquired images using a Two-photon super-resolution point scanning confocal microscope (Nikon, Japan) and selected representative images for each sample. Twenty cells from three independent biological experiments were randomly set for statistical analysis.

### Statistical analysis and reproducibility

All statistical procedures were performed using SPSS 6.0 software (IBM, USA). For more than two groups, statistical significance was determined using one-way analysis of variance (ANOVA), followed by Bonferroni’s multiple comparison tests. For two groups, the independent samples were subjected to Student’s *t* tests (two-tailed). Statistical significance was defined as *p < 0.05, **p < 0.01, or ****p < 0.001. For correlation analysis, linear correlation between variables was tested by calculating the Pearson’s correlation coefficient.

For qRT-PCR and cytotoxicity assays, we conducted three independent biological experiments and six replicates were set for each individual experiment. All the western blots and fluorescence tests were conducted for at least three independent biological experiments and the representative images are shown.

## Supplementary information

Supplemental figure legends

Supplemental figure 1

Supplemental figure 2

Supplemental figure 3

Supplemental figure 4

Supplemental figure 5

Supplemental figure 6

Supplemental figure 7

Supplemental figure 8

Supplemental figure 9

Supplemental figure 10

Supplemental figure 11

Supplemental figure 12

Supplemental figure 13

Supplemental figure 14

## Data Availability

All data supporting the findings of this study are available from the corresponding author on reasonable request.
