# Supplementary material for: Cell fate determined by the activation balance between PKR and SPHK1
Source: Cell Death Differ. 2020 Aug 15;28(1):401–18. doi: 10.1038/s41418-020-00608-8 (PMC7852545; doi:10.1038/s41418-020-00608-8)
Supplement: Supplementary file 1 — Supplemental figure legends [file 41418_2020_608_MOESM1_ESM.docx]

**Supplementary figure legends**

**Fig S1** The HepG2 and HEK293T cells were tested following treatment with various concentrations of DON for 24 h. The relative cell viability levels were detected by CCK-8 assay and normalized to those of control (n = 6).

**Fig S2** The levels of phosphorylated PKR and SPHK1 were evaluated following treatment with 400 nM or 2 μM DON for the indicated time-periods in HEK293T and HepG2 cells. All the target protein levels were analyzed by western blotting.

**Fig S3 a** ER stress-related protein expression levels were evaluated following treatment with 2 μM DON for 3 h in HepG2 and HEK293T cells. **b** ER stress-related protein expression levels were evaluated following treatment with 10 ng/ml TNF‐α for 3 h in HepG2 and HEK293T cells. All the target protein levels were analyzed by western blotting.

**Fig S4 a** Targeted knockout efficiency of AsCpf1-*PKR*-sgRNA was evaluated by nucleic acid electrophoresis detection. Cells transfected with the empty plasmid PY30 served as the positive control. **b,** A sketch depicting the gene knockout regions in *PKR* genome**. c** Alignment of sequencing result at genome locations of AsCpf1-*PKR*-sgRNA (starting with the first exon).

**Fig S5** The levels of phosphorylated PKR were evaluated following treatment with various concentrations of PolyI:C for 3 h in HEK293T cells. All the target protein levels were analyzed by western blotting.

**Fig S6 a** The levels of SPHK1 phosphorylation were evaluated in PKR knockdown cells. HEK293T control and PKR knockdown cells at 90% confluence were harvested and subjected to western blotting analysis. The exposure time for phosphorylated eIF2α, IRE1α, XBP1(s) and CHOP were 6s (the endogenous exposure time for these proteins were 2 s). **b, c** ER stress-related protein levels during DON or TNF‐α treatment were evaluated in PKR knockdown cells. HEK293T control and PKR knockdown cells at 70% confluence were incubated with 400 nM DON or 10 ng/mL TNF‐α for 3 h. The cells were then harvested and subjected to western blotting analysis. **d** ER stress-related protein levels during PolyI:C treatment were evaluated in PKR knockout cells. HEK293T control and PKR knockout cells at 70% confluence were incubated with 10 μg/mL PolyI:C for 3 h. The cells were then harvested and subjected to western blotting analysis.

**Fig S7 a** Cytotoxicity of DON was evaluated in PKR knockdown cells. HEK293T control and PKR knockdown cells at 70% confluence were treated with various concentrations of DON for 24 h. Cell viability was then determined with CCK-8 (n = 6). **b** Cell apoptosis resulting from TNF-α treatment in PKR knockdown cells. HEK293T control and PKR knockdown cells were evaluated with 10 ng/mL TNF-α by targeting cleaved caspase 9. The cells were then harvested and subjected to western blotting analysis.

**Fig S8** The effect of IKKα and phosphorylated MAPKs on the cytotoxicity of DON. HepG2 cells were pre-incubated with P38 inhibitor (10 μM SB203580), ERK1/2 inhibitor (0.5 μM SCH772984), JNK inhibitor (10 μM SP600125) or IKKα inhibitor (2 μM IKK-16) for 1 h, and then, the cells were incubated with 2 μM DON for 24 h, followed by detection of cell viability with CCK-8 (n = 6). Statistical significance was defined as *P* < 0.05 (*), *P* < 0.01 (**), or *P* < 0.001 (***).

**Fig S9 a** The levels of SPHK1 phosphorylation were evaluated in PKR knockdown cells. HEK293T control and PKR knockdown cells at 90% confluence were harvested and subjected to western blotting analysis. **b** The levels of phosphorylated SPHK1 during PolyI:C treatment were evaluated in PKR knockout cells. HEK293T control and PKR knockout cells at 70% confluence were incubated with 10 μg/mL PolyI:C for 3 h. The cells were then harvested and subjected to western blotting analysis. **c, d** The expression levels of phosphorylated SPHK1 were evaluated following treatment with DON or TNF‐α in PKR knockdown cells. HEK293T control and PKR knockdown cells at 70% confluence were incubated with 400 nM DON or 10 ng/mL TNF-α for 3 h. The cells were then harvested and subjected to western blotting analysis.

**Fig S10** Phosphorylation of eIF2α by PKR was evaluated using γ-^32^P ATP labeling analysis. Various concentrations of cell lysates from transfected Flag tagged-PKR were purified using Flag (M2) magnetic beads and mixed with equal aliquot of GST-eIF2α. The ^32^P labeling kinase reactions were conducted in the presence of 0.5 μCi γ-^32^P ATP and subjected to SDS-PAGE, and then, the ^32^P incorporation were transferred to a phosphor screen and developed by PerkinElmer scanner.

**Fig S11** **a** Various concentrations of purified GST-PKR were mixed with equal aliquots of purified GST-eIF2α. Phosphorylation status of eIF2α was examined by specific anti-p-eIF2α antibody. **b** PolyI:C was used to activate PKR kinase activities *in vitro*, and then, the kinase activities were measured using the ADP-Glo kinase assay. P represents PKR and PIC represents PolyI:C. **c** Various concentrations of purified GST-SPHK1 were mixed with equal aliquot of purified GST-PKR, and then, the kinase activities were measured using the ADP-Glo kinase assay. S represents SPHK1.

**Fig S12** Effects of SPHK1 overexpression on PACT-PKR interaction. 0, 1.5 or 3 μg SPHK1-Flag were transfected into HEK293T cells and processed for immunoprecipitation, and then, the precipitates and whole cell lysates were then analyzed by western blotting.

**Fig S13** The localization of SPHK1 was evaluated with DON or TNF‐α treatment in PKR knockout cell line by an immunofluorescence assay. HEK293T control and PKR knockout cells were treated with 400 nM DON or 10 ng/mL TNF-α for 3 h, and then the cells were fixed and stained with anti-SPHK1 antibodies (green) and DAPI (blue) (scale bar=50 μm).

**Fig S14 a** Statistical results of fluorescence intensity of SPHK1 and PKR combinations in BiFC assay. **b** The effect of the position of amino acid S225 in SPHK1 on SPHK1–PKR interaction. 225A/225D HA-tagged SPHK1 and an equivalent amount of Flag-tagged PKR were transfected into HEK293T cells and processed for immunoprecipitation. The precipitates and whole-cell lysates were then analyzed by western blotting.
