# Supplementary figures and images for: Cell fate determined by the activation balance between PKR and SPHK1
Source: Cell Death Differ. 2020 Aug 15;28(1):401–18. doi: 10.1038/s41418-020-00608-8 (PMC7852545; doi:10.1038/s41418-020-00608-8)

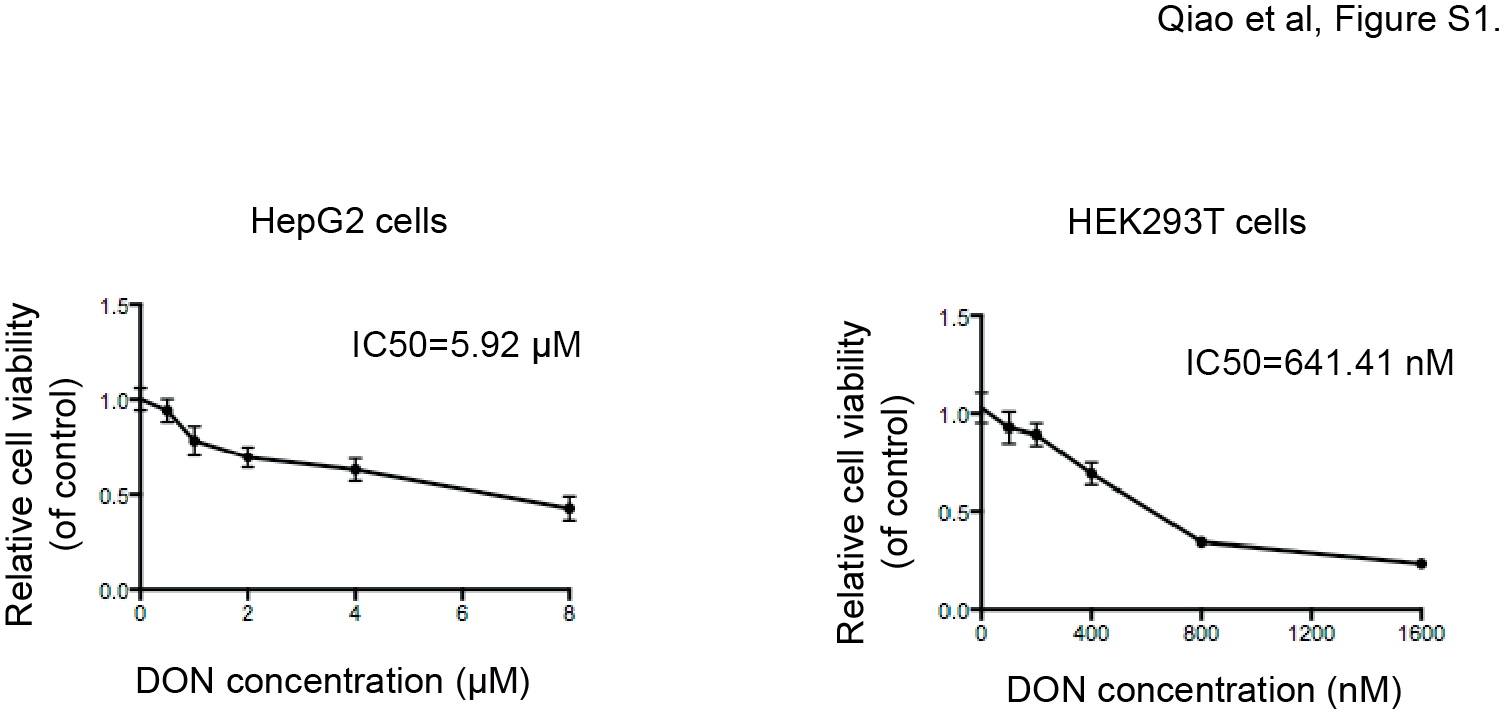

Supplement: Supplementary file 2 — Supplemental figure 1 [file 41418_2020_608_MOESM2_ESM.png]

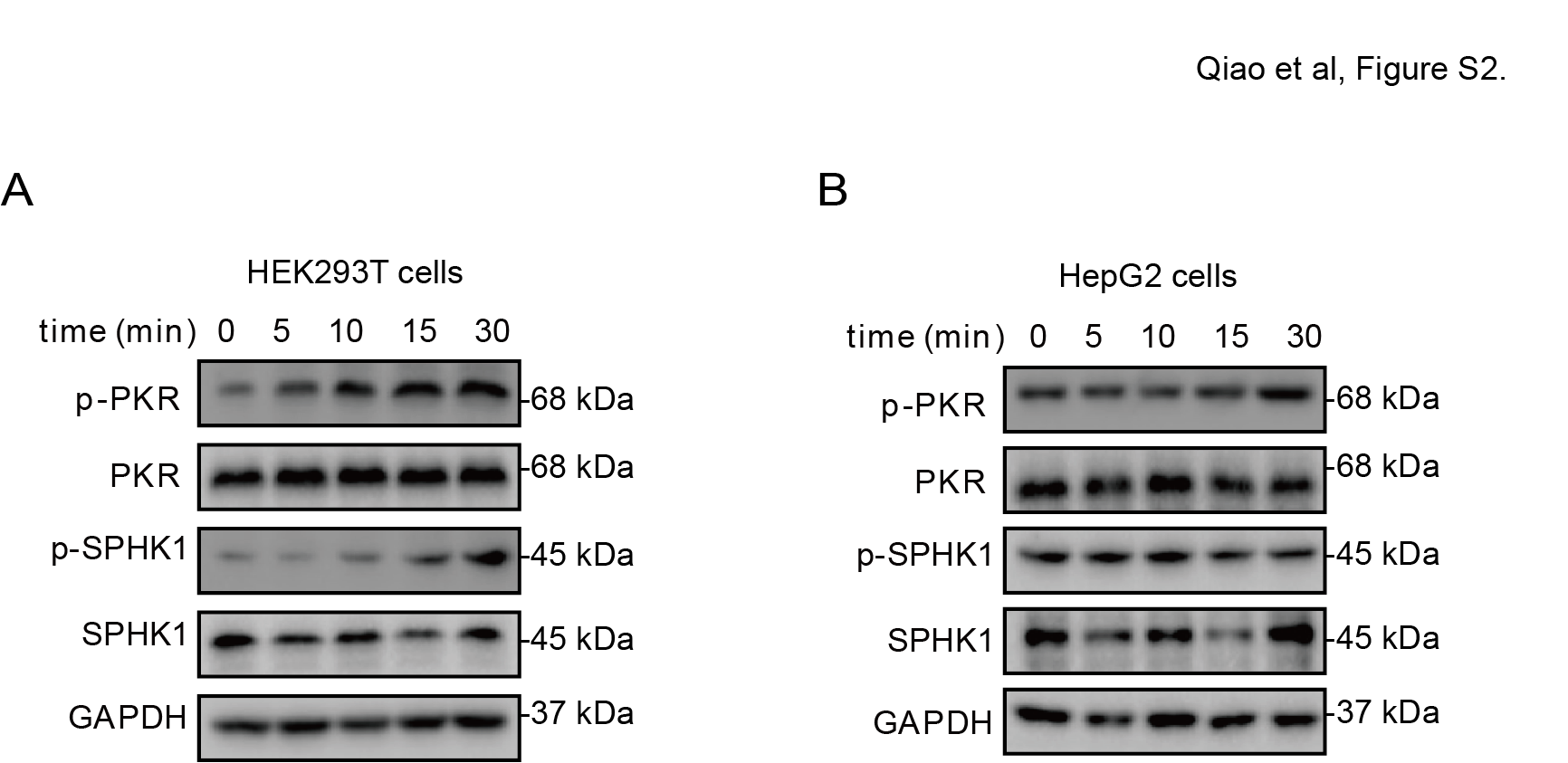

Supplement: Supplementary file 3 — Supplemental figure 2 [file 41418_2020_608_MOESM3_ESM.png]

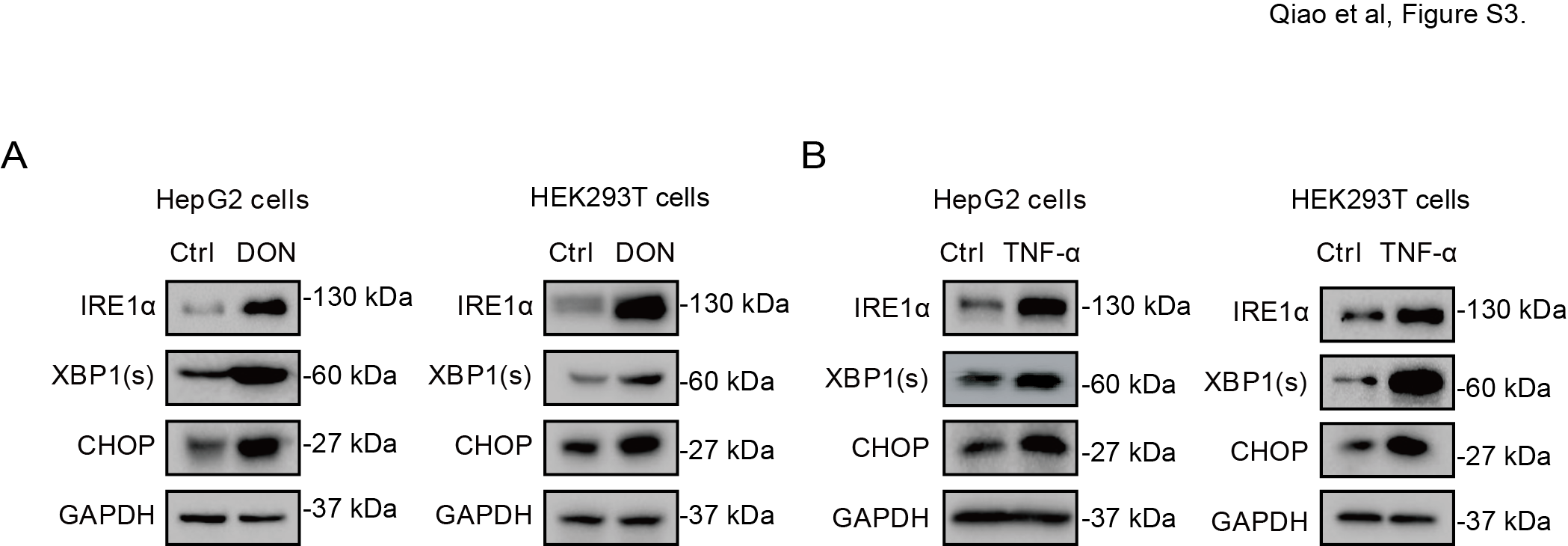

Supplement: Supplementary file 4 — Supplemental figure 3 [file 41418_2020_608_MOESM4_ESM.png]

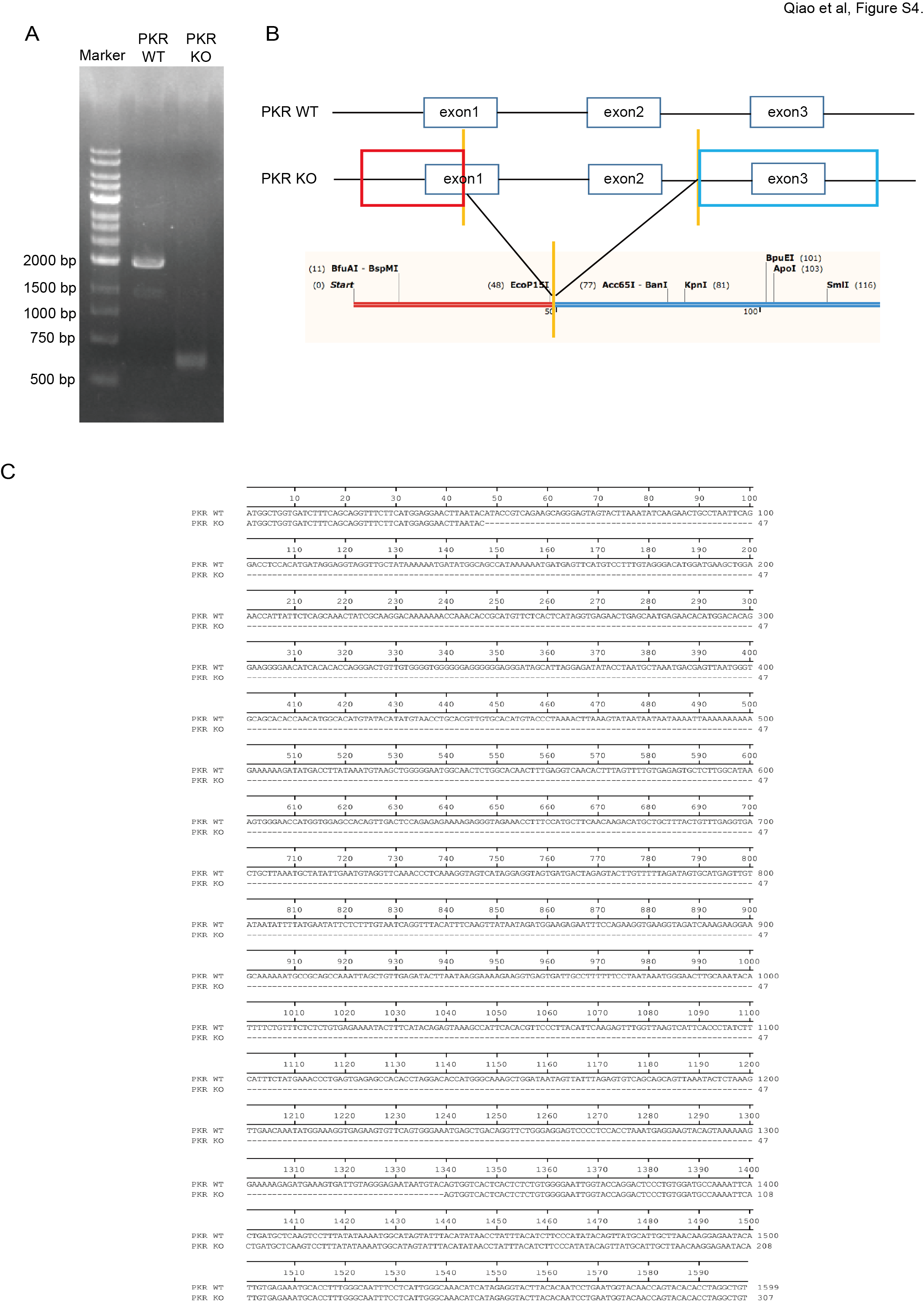

Supplement: Supplementary file 5 — Supplemental figure 4 [file 41418_2020_608_MOESM5_ESM.png]

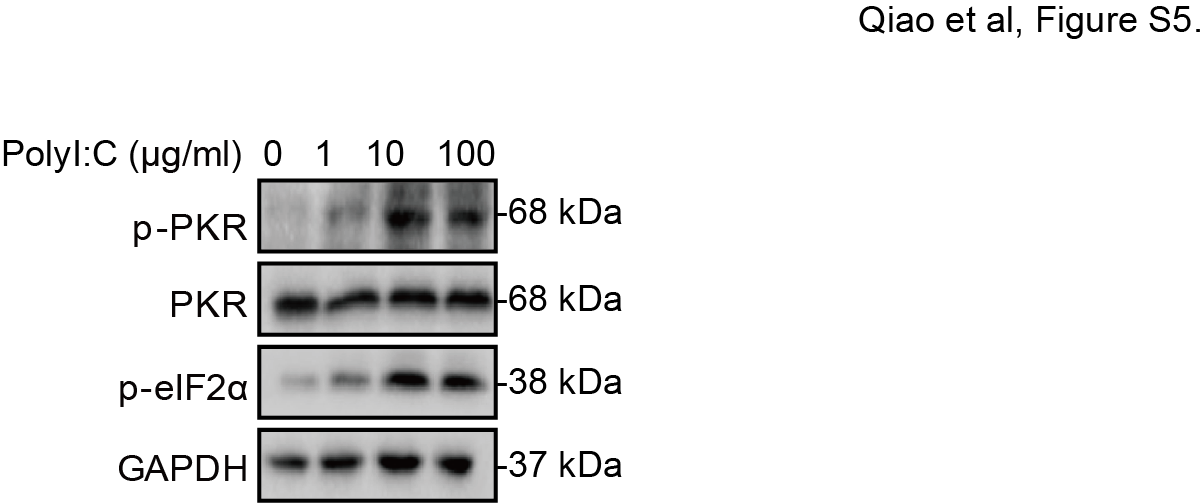

Supplement: Supplementary file 6 — Supplemental figure 5 [file 41418_2020_608_MOESM6_ESM.png]

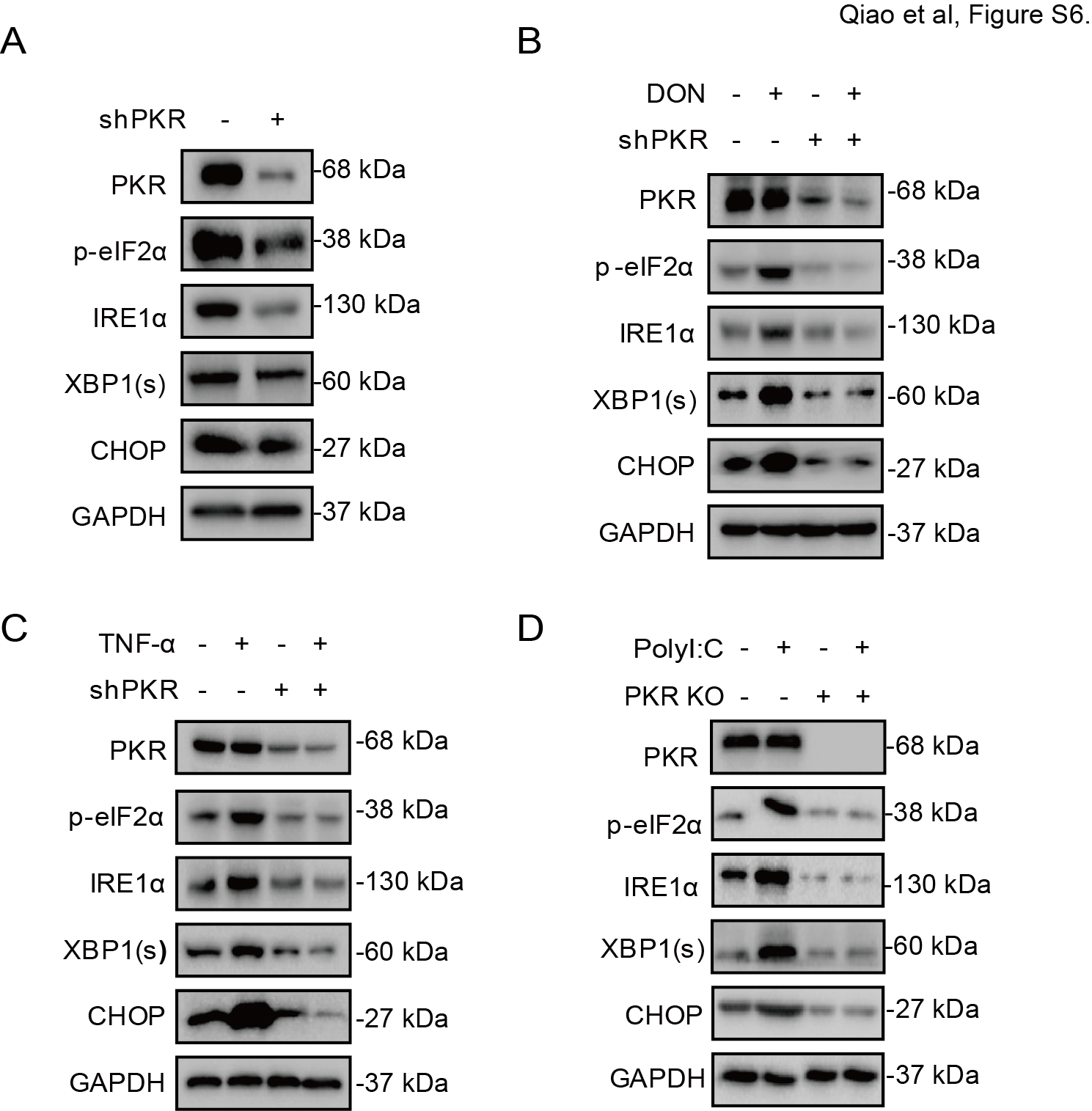

Supplement: Supplementary file 7 — Supplemental figure 6 [file 41418_2020_608_MOESM7_ESM.png]

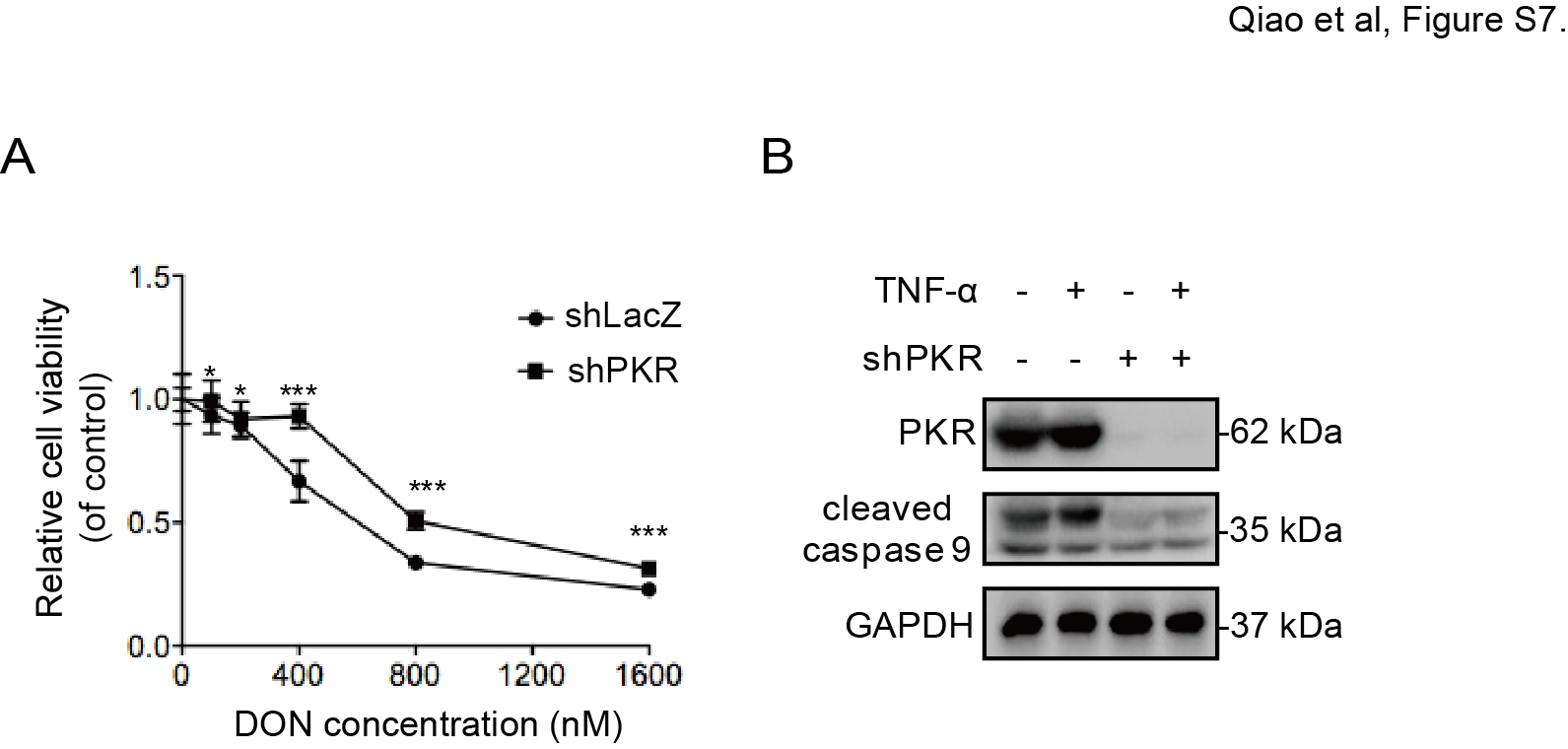

Supplement: Supplementary file 8 — Supplemental figure 7 [file 41418_2020_608_MOESM8_ESM.png]

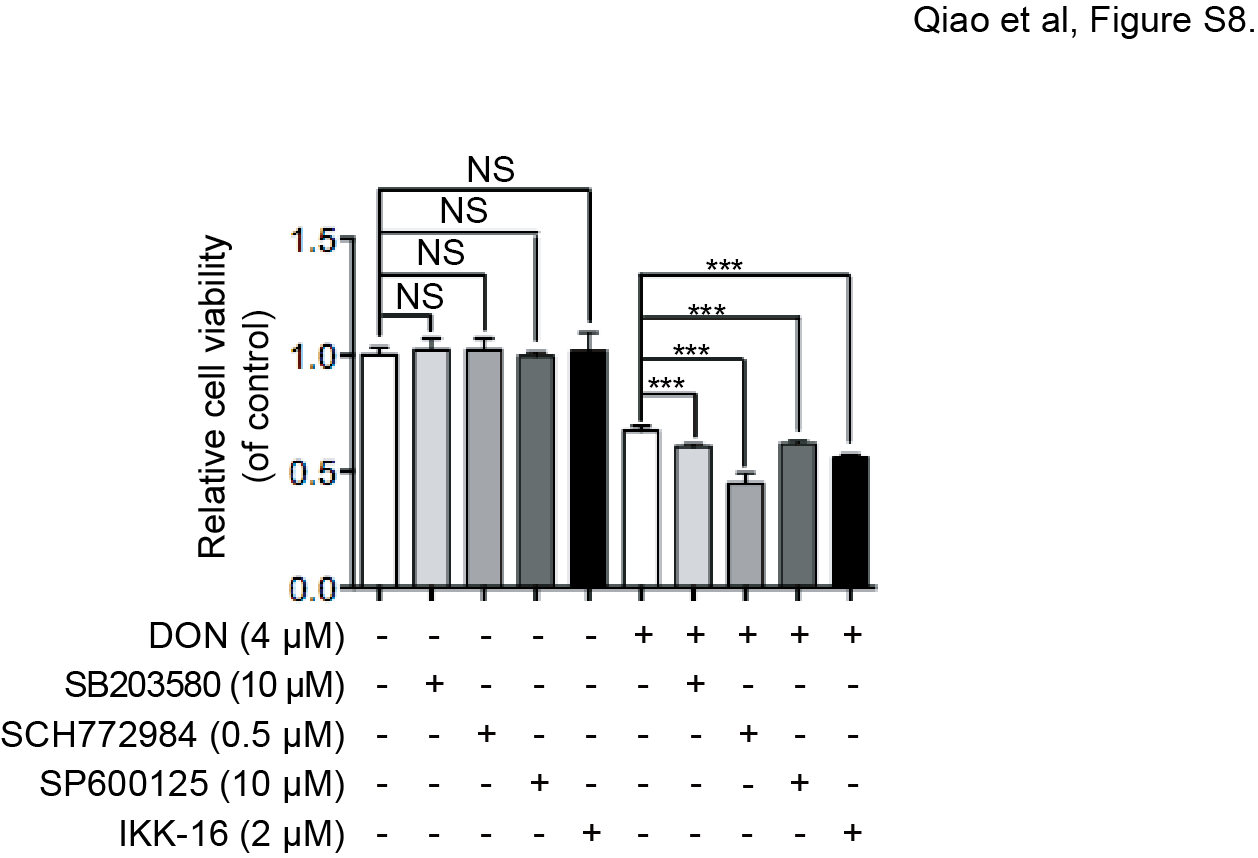

Supplement: Supplementary file 9 — Supplemental figure 8 [file 41418_2020_608_MOESM9_ESM.png]

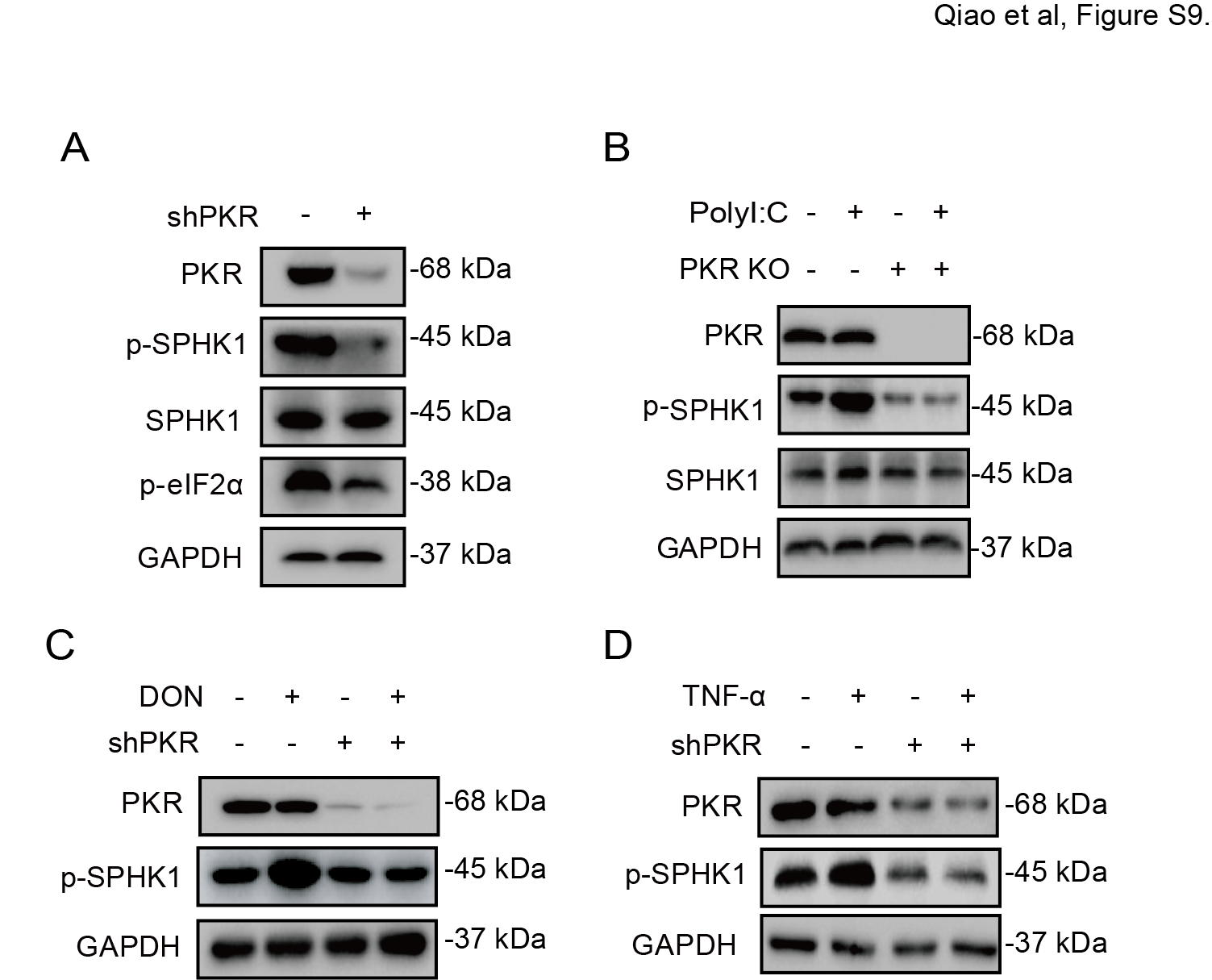

Supplement: Supplementary file 10 — Supplemental figure 9 [file 41418_2020_608_MOESM10_ESM.png]

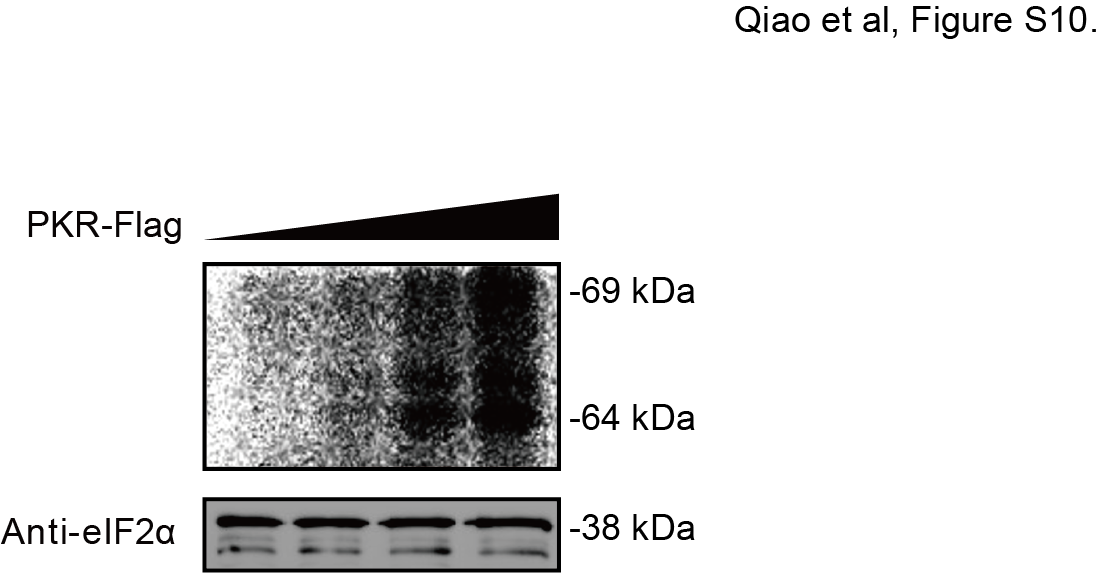

Supplement: Supplementary file 11 — Supplemental figure 10 [file 41418_2020_608_MOESM11_ESM.png]

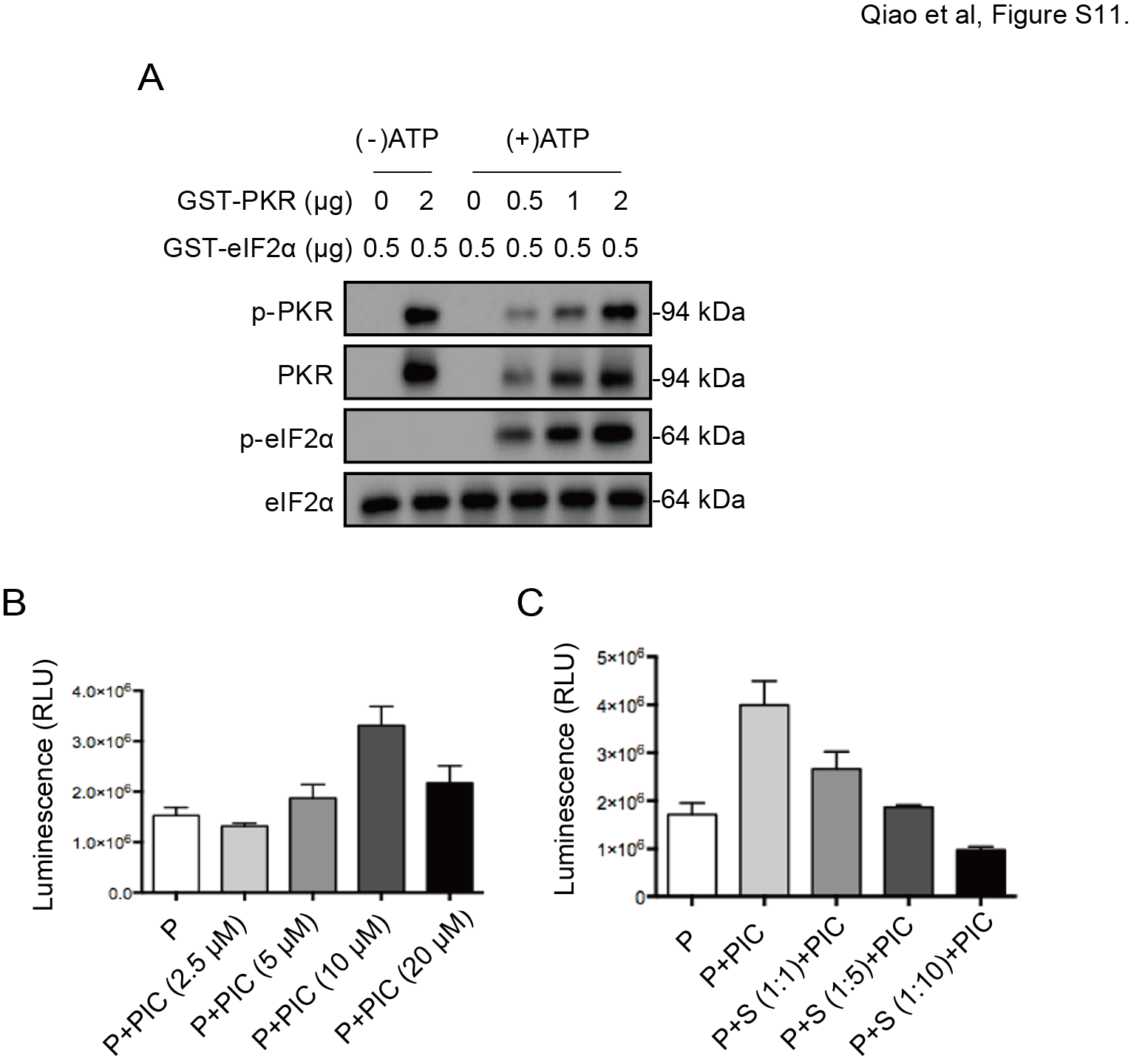

Supplement: Supplementary file 12 — Supplemental figure 11 [file 41418_2020_608_MOESM12_ESM.png]

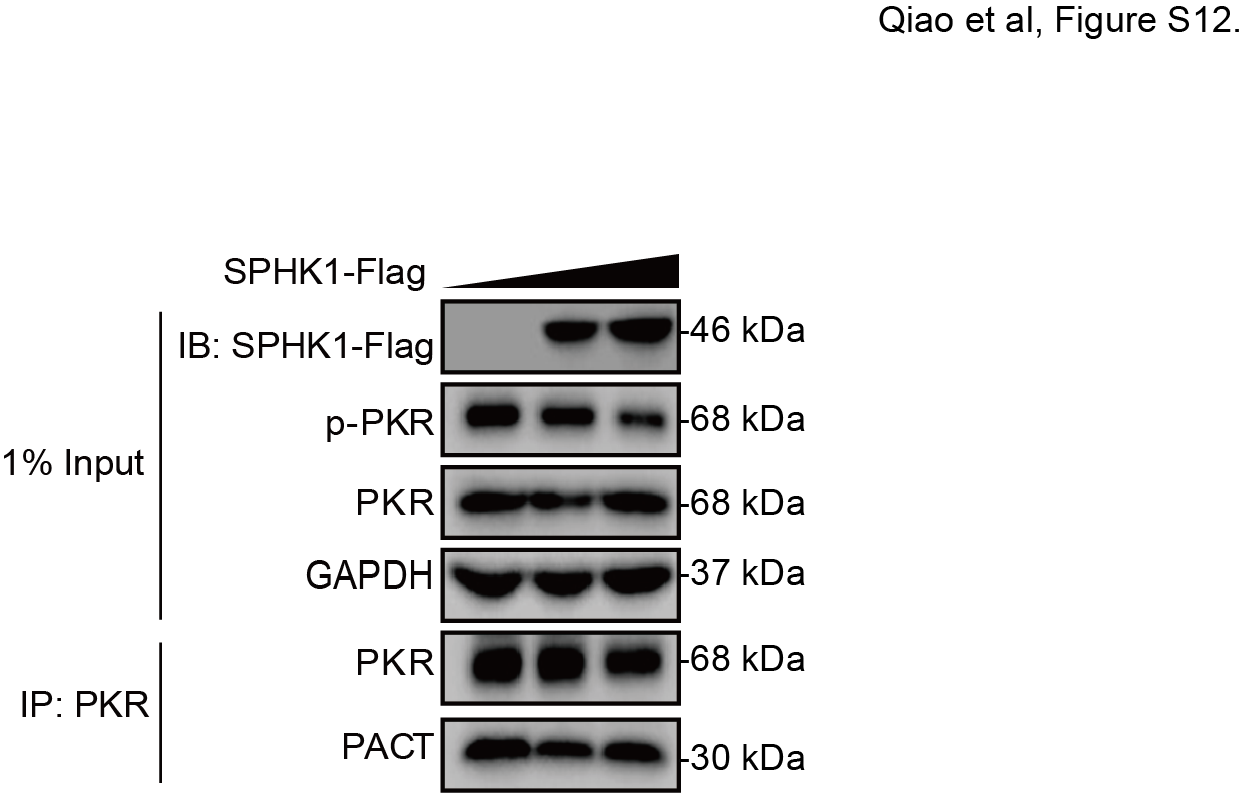

Supplement: Supplementary file 13 — Supplemental figure 12 [file 41418_2020_608_MOESM13_ESM.png]

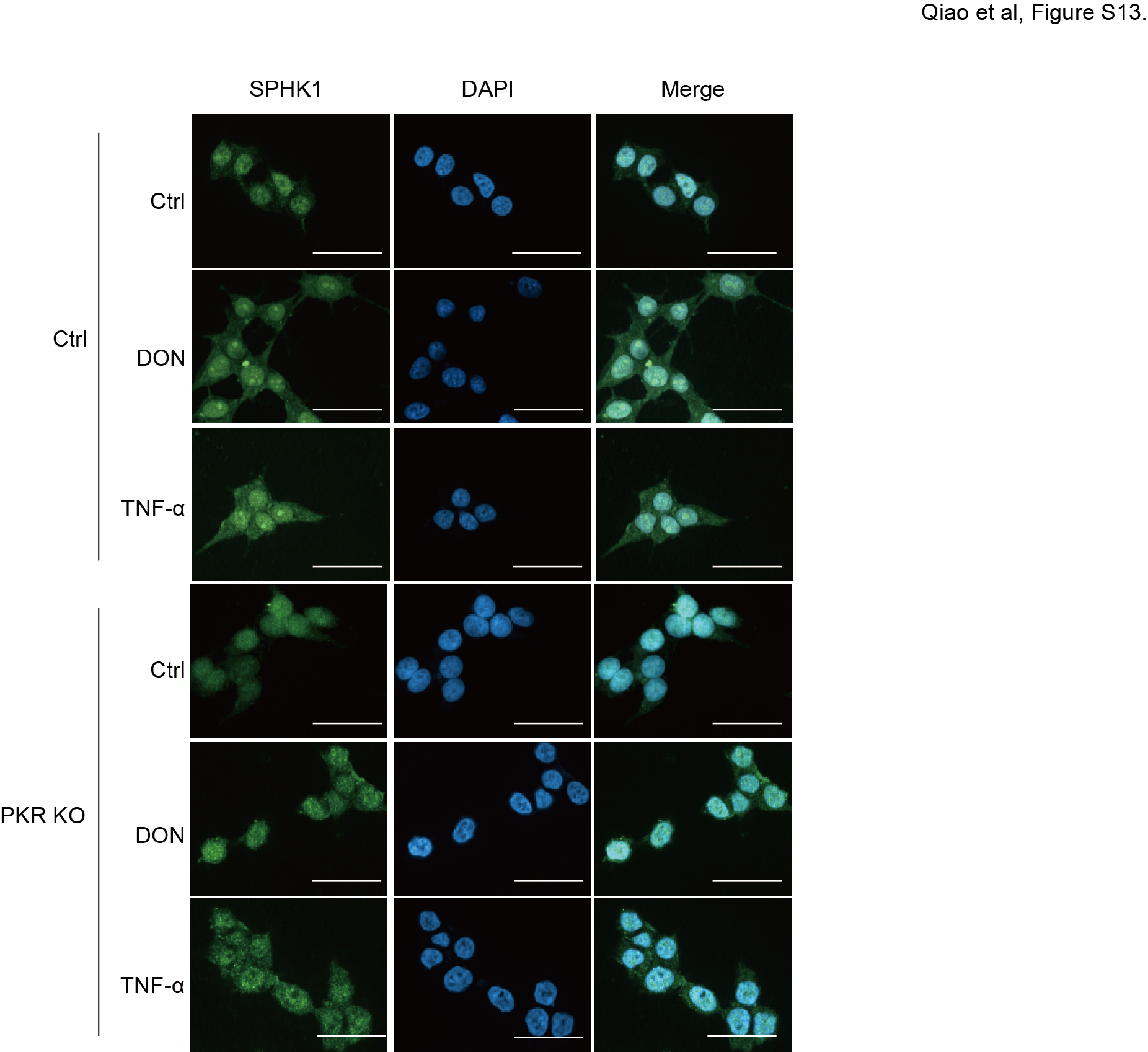

Supplement: Supplementary file 14 — Supplemental figure 13 [file 41418_2020_608_MOESM14_ESM.png]

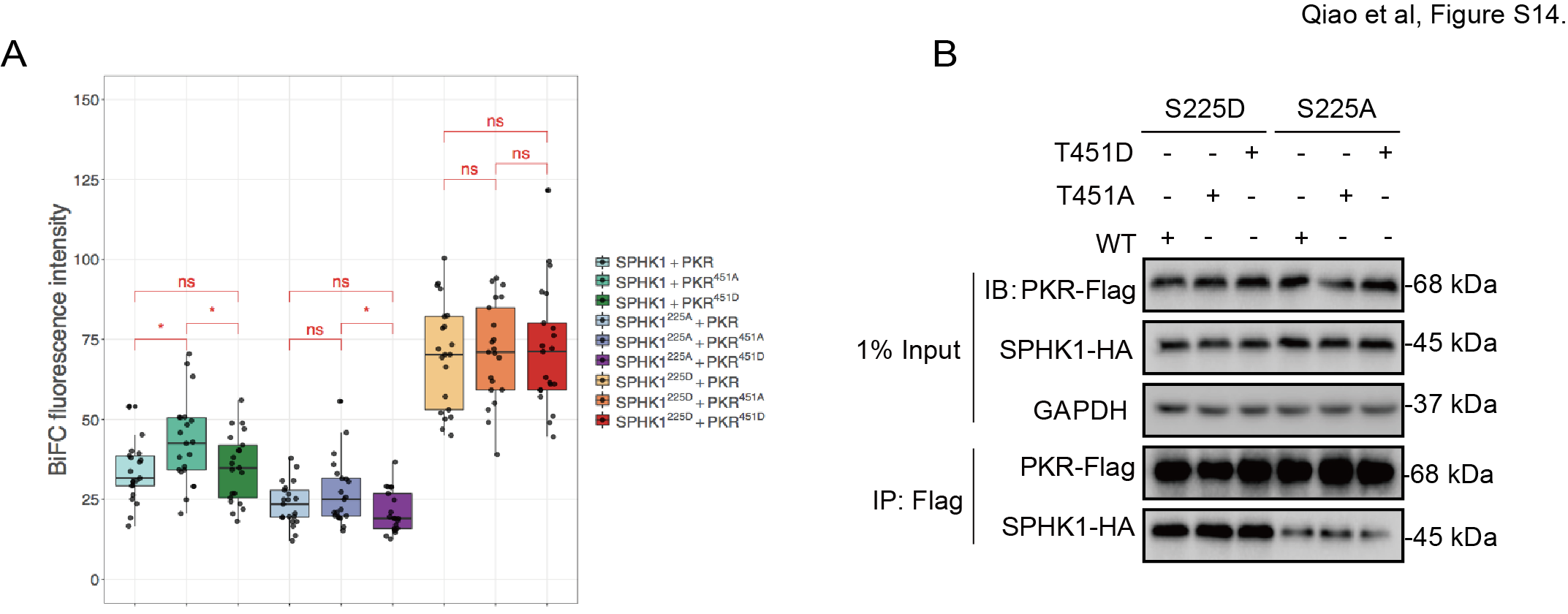

Supplement: Supplementary file 15 — Supplemental figure 14 [file 41418_2020_608_MOESM15_ESM.png]
